# New Prenylated Indole Homodimeric and Pteridine Alkaloids from the Marine-Derived Fungus *Aspergillus austroafricanus* Y32-2

**DOI:** 10.3390/md19020098

**Published:** 2021-02-09

**Authors:** Peihai Li, Mengqi Zhang, Haonan Li, Rongchun Wang, Hairong Hou, Xiaobin Li, Kechun Liu, Hao Chen

**Affiliations:** 1 Engineering Research Center of Zebrafish Models for Human Diseases and Drug Screening of Shandong Province, Shandong Provincial Engineering Laboratory for Biological Testing Technology, Biology Institute, Qilu University of Technology (Shandong Academy of Sciences), Jinan 250103, China; liph@sdas.org (P.L.); mengqi@sdas.org (M.Z.); 17862958363@163.com (H.L.); wangrc@sdas.org (R.W.); houhr@sdas.org (H.H.); 2 State Key Laboratory of Biobased Material and Green Papermaking, Qilu University of Technology (Shandong Academy of Sciences), Jinan 250353, China; 3 Key Laboratory for Biosensor of Shandong Province, Biology Institute, Qilu University of Technology (Shandong Academy of Sciences), Jinan 250103, China; 4 Key Laboratory of Marine Bioactive Substances, First Institute of Oceanography, Ministry of Natural Resources, Qingdao 266061, China

**Keywords:** marine-derived fungus, *Aspergillus austroafricanus*, novel bioactive metabolites, pro-angiogenesis, anti-inflammatory effects

## Abstract

Chemical investigation of secondary metabolites from the marine-derived fungus *Aspergillus austroafricanus* Y32-2 resulted in the isolation of two new prenylated indole alkaloid homodimers, di-6-hydroxydeoxybrevianamide E (**1**) and dinotoamide J (**2**), one new pteridine alkaloid asperpteridinate A (**3**), with eleven known compounds (**4**–**14**). Their structures were elucidated by various spectroscopic methods including HRESIMS and NMR, while their absolute configurations were determined by ECD calculations. Each compound was evaluated for pro-angiogenic, anti-inflammatory effects in zebrafish models and cytotoxicity for HepG2 human liver carcinoma cells. As a result, compounds **2**, **4**, **5**, **7**, **10** exhibited pro-angiogenic activity in a PTK787-induced vascular injury zebrafish model in a dose-dependent manner, compounds **7**, **8**, **10**, **11** displayed anti-inflammatory activity in a CuSO_4_-induced zebrafish inflammation model, and compound **6** showed significant cytotoxicity against HepG2 cells with an IC_50_ value of 30 µg/mL.

## 1. Introduction

The ocean has the characteristics of high salinity, high pressure, low temperature, low oxygen content, and oligotrophic environment, which enables microorganisms to have unique metabolic adaptation mechanisms and produce natural products with novel structures and diverse bioactivities [1]. Marine-derived fungi have been found to be a rich source of natural products due to their complex genetic background and abundant metabolites [2]. In recent years, a large number of novel secondary metabolites, such as polyketides, alkaloids, terpenes, steroids, peptides, etc., have been discovered from marine-derived *Aspergillus* species [3], and showed diverse bioactivities like antibacterial, antitumor, antioxidant, and anti-inflammatory activities [4]. More than 80% natural products were directly or indirectly related to small molecule drugs for the treatment of various diseases in the last 30 years, and many marine alkaloids with bioactivities have been comprehensively studied for drug development [5,6].

**Figure 1:**
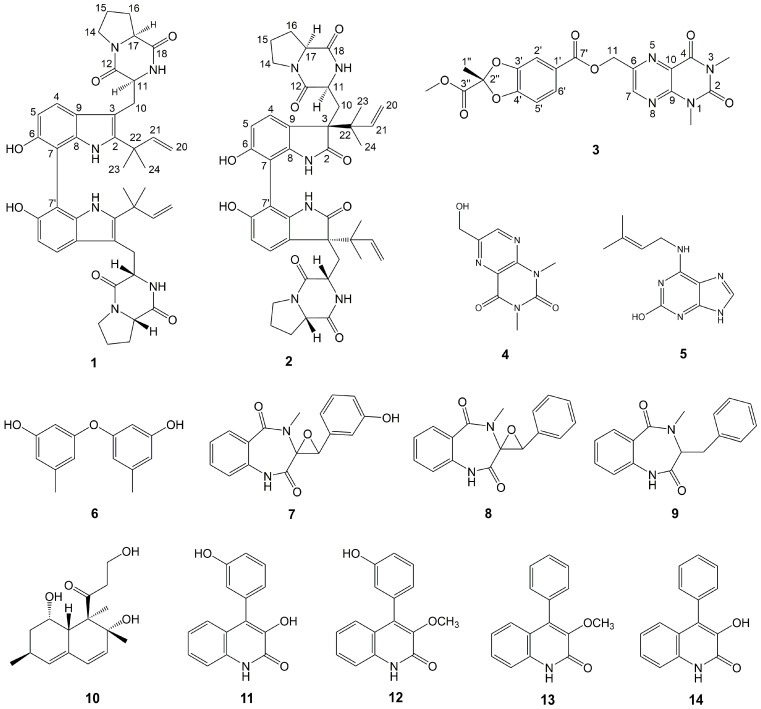
Structures of Compounds **1**–**14**.

In our previous study, a series of fungal secondary metabolites were isolated and characterized with antitumor or cardiovascular effects [7,8]. To discover more natural products with pharmacological activities from marine-derived fungi, the fungal strain *Aspergillus austroafricanus* Y32-2 has been isolated from a seawater sample collected from the Indian Ocean. Chemical investigation of the secondary metabolites of Y32-2 fermented on rice medium resulted in the isolation of fourteen compounds, including two new prenylated indole alkaloid homodimers and one new pteridine alkaloid, named di-6-hydroxydeoxybrevianamide E (**1**), dinotoamide J (**2**) and asperpteridinate A (**3**), along with eleven known compounds (**4**–**14**) [7,9,10,11,12,13,14,15,16] (Figure 1). Among them, compound **4** was isolated for the first time as a natural product. 

The prenylated indole alkaloids contain a bicyclo[2.2.2]diazaoctane or diketopiperazine ring, and has been reported to have antitumor, antibacterial, and insecticidal activities [17]. Here two new prenylated indole alkaloid homodimers and other isolated compounds were all tested for pro-angiogenic and anti-inflammatory effects in zebrafish models and cytotoxicity towards HepG2 human liver carcinoma cells. Compounds **2**, **4**, **5**, **7**, and **10** exhibited angiogenesis promoting activity in a dose-dependent manner. Compounds **7**, **8**, **10**, and **11** also displayed anti-inflammatory activity in a dose-dependent manner. In addition, compound **6** showed cytotoxicity against HepG2 cells. In this paper, the isolation, structure elucidation, and bioactivity of all isolated compounds are reported.

## 2. Results and Discussion

### 2.1. Structure Elucidation

**Figure 2:**
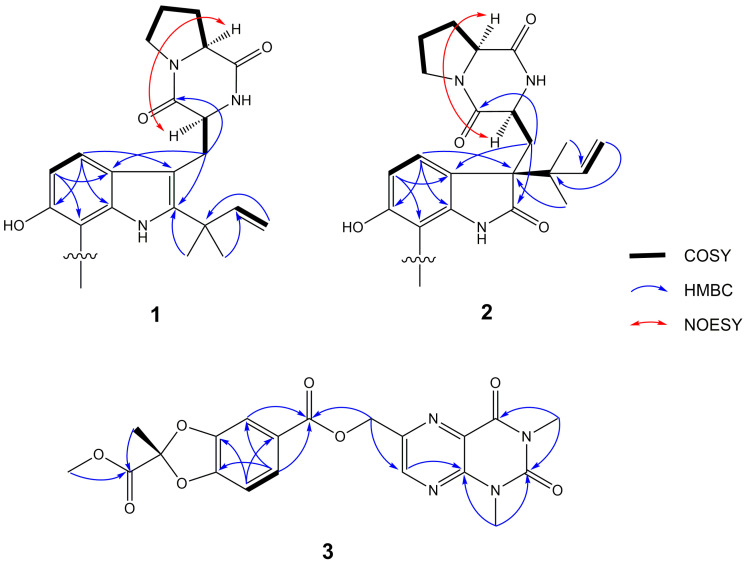
The ^1^H, ^1^H-correlation spectroscopy (^1^H, ^1^H-COSY), key heteronuclear multiple-bond correlation spectroscopy (HMBC) and nuclear overhauser effect spectroscopy (NOESY) correlations of compounds **1**, **2** (only half showed) and **3**.

**Figure 3:**
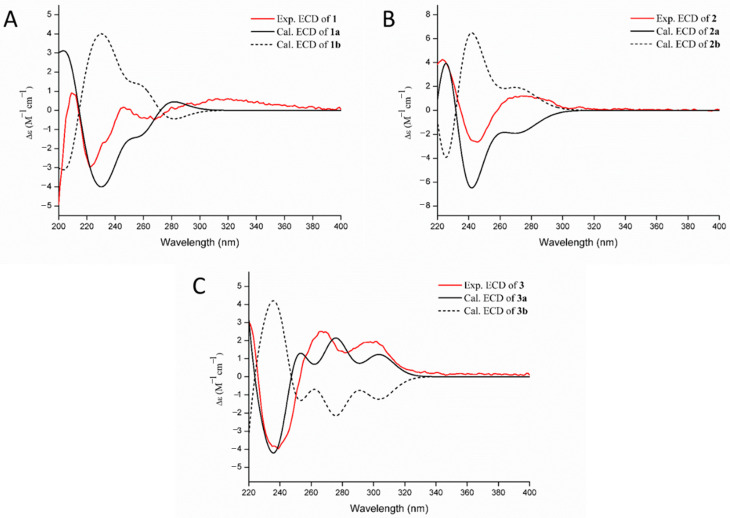
Measured CD and calculated equivalent circulating density (ECD) curves of compounds **1** (**A**), **2** (**B**) and **3** (**C**).

Compound **1**, obtained as yellow amorphous powder, possessed a molecular formula of C_42_H_48_N_6_O_6_ by the negative HR-ESI-MS (*m/z* 731.3559 [M − H]^−^, calculated 731.3557), requiring 22 unsaturations. The HPLC chromatographic behavior of **1** was unusual and existed always as a 1:1 inseparable mixture. Many of the NMR signals also appeared in pairs, hinting towards structural distinctiveness and complexity. The ^1^H NMR spectrum (Table 1) in DMSO-*d*_6_ of **1** showed two pairs of mutually coupled aromatic protons at *δ*_H_ 7.35, 7.37 (each H, d, *J* = 8.4 Hz) and 6.80, 6.81 (each H, d, *J* = 8.4 Hz), a set of vinyl proton signals at *δ*_H_ 6.15, 6.17 (each H, dd, *J* = 17.5, 10.7 Hz), 5.06 (2H, br d, *J* = 17.5 Hz) and 5.01 (2H, br d, *J* = 10.7 Hz), four methyl singlets at *δ*_H_ 1.42 (12H, s), as well as six active hydrogen signals at *δ*_H_ 9.09 (2H, br s), 8.45, 8.58 (each H, s) and 6.21, 6.30 (each H, s). The ^13^C NMR spectrum (Table 1) showed four amidocarbonyl carbon signals at *δ*_C_ 169.3, 169.4 (C-18, 18′) and 165.6 (C-12, 12′, overlapped), twenty aromatic or olefinic carbon signals, containing four vinyl carbons at *δ*_C_ 146.4, 146.5 (C-21, 21′) and 111.29, 111.33 (C-20, 20′), and four nitrogen-bearing methine signals at *δ*_C_ 58.5 (C-17, 17′, overlapped) and 55.0, 55.3 (C-11, 11′). The above NMR features were similar to those of 6-hydroxydeoxybrevianamide E [18], a cyclic dipeptide produced by *Aspergillus* and *Penicillium* species, and a careful and rigorous analysis of the ^1^H, ^1^H-COSY and HMBC correlations (Figure 2) also supported this inference. However, there were two obvious differences in their NMR signals: (1) different substitution patterns on the indole ring; (2) most of the NMR signals in **1** appeared in pairs. Based on the HSQC and HMBC correlation, the C-7, 7′ (*δ*_C_ 104.0, 104.1) were aromatic quaternary carbon signals that were different from 6-hydroxydeoxybrevianamide E, confirmed that the positions C-7, 7′ of the indole ring were substituted. Considering its molecular formula, compound **1** was deduced as a dimer of 6-hydroxydeoxybrevianamide E via C-7 and C-7′. Due to a certain steric hindrance, the structure existed as a 1:1 mixture of inseparable rotamers. The relative configuration of the cyclic dipeptide moiety was determined by the NOESY correlation (Figure 2) of H-11 and H-17. Based on the relative configuration, two probable forms of its absolute configuration, 1a (11*S*, 17*S*, 11′*S*, 17′*S*) and 1b (11*R*, 17*R*, 11′*R*, 17′*R*), were respectively used for the ECD calculations, and the absolute configuration was assigned as 11*S*, 17*S*, 11′*S*, 17′*S* (Figure 3), which was in consistent with that of 6-hydroxydeoxybrevianamide E. Therefore, the structure of **1** was unequivocally established as shown in Figure 1 and named as di-6-hydroxydeoxybrevianamide E.

Compound **2** was obtained as a yellow amorphous powder. The molecular formula was determined to be C_42_H_48_N_6_O_8_ by the negative HRESIMS (*m/z* 763.3440 [M − H]^−^, calculated 763.3456), indicating 22 degrees of unsaturation. The NMR spectra (Table 1) of **2** revealed 24 proton and 21 C-atom signals, suggesting **2** to be a symmetrical homodimer. The ^1^H NMR spectrum for **2** showed two aromatic proton signals at *δ*_H_ 7.11 (1H, d, *J* = 8.2 Hz) and 6.39 (1H, d, *J* = 8.2 Hz), three vinyl proton signals at *δ*_H_ 4.94 (1H, br d, *J* = 17.5 Hz), 5.00 (1H, br d, *J* = 10.9 Hz) and 6.15 (1H, dd, *J* = 17.5, 10.9 Hz), two methyl singlets at *δ*_H_ 0.96 (3H, s), 0.98 (3H, s), as well as three active hydrogen signals at *δ*_H_ 7.57 (1H, s), 9.28 (1H, br s), and 9.31 (1H, s). The ^13^C NMR data (Table 1) revealed the presence of three carbonyl carbon signals at *δ*_C_ 180.1 (C-2), 169.8 (C-18) and 165.6 (C-12), eight aromatic or olefinic carbon signals containing two vinyl carbons at *δ*_C_ 143.9 (C-21) and 112.6 (C-20), and two nitrogen-bearing methines at *δ*_C_ 58.3 (C-17) and 52.6 (C-11). Extensive comparison of the above NMR spectra with those of notoamide J [17] revealed that both structures were very similar, except for the substitution patterns on the C-7 position of indole ring. Considering its molecular formula, compound **2** was also identified as a homodimer of notoamide J via C-7 and C-7′. Due to one single signal set in the NMR spectrum, one single peak in the chiral column chromatography and less steric hindrance in the structure than compound **1**, it was deduced to be a freely rotating homologous dimer. With the aid of the ^1^H, ^1^H-COSY, HSQC and HMBC correlations, the planar structure of **2** was established as shown (Figure 1). The relative configuration of the cyclic dipeptide moiety was deduced by a NOESY correlation between H-11 and H-17, suggested that both protons had the same co-facial orientation. Because of the similar NMR data between **2** and notoamide J, the relative configuration of the positions C-3, C-11 and C-17 were determined to be similar to that of notoamide J [17]. By comparison of the experimental and calculated ECD spectra of **2**, the absolute configuration was tentatively assigned as 3*R*, 11*S*, 17*S*, 3′*R*, 11′*S*, and 17′*S* (Figure 3), which was also probably verified by the identical CD spectrum between **2** and notoamide J. So, the structure of **2** was tentatively assigned as shown in Figure 1 and named as dinotoamide J.

Asperpteridinate A was obtained as a yellow amorphous powder. The molecular formula C_20_H_18_N_4_O_8_ was assigned on the basis of the HRESIMS peak at *m*/*z* 465.1018 [M + Na]^+^ (calcd. 465.1023), requiring 14 degrees of unsaturation. The ^1^H NMR spectrum of **3** showed the signals for a 1,2,4-trisubstituted benzene ring system at *δ*_H_ 7.49 (1H, d, *J* = 1.4 Hz), 7.10 (1H, d, *J* = 8.3 Hz) and 7.65 (1H, dd, *J* = 8.3, 1.4 Hz), one vinyl proton at *δ*_H_ 8.94 (1H, s), three O-methyl or N-methyl at *δ*_H_ 3.74 (3H, s), 3.53 (3H, s), 3.31 (3H, s), one O-methylene at *δ*_H_ 5.49 (2H, s), one methyl at *δ*_H_ 1.90 (3H, s). The ^13^C NMR data (Table 2) revealed the presence of four carbonyl at *δ*_C_ 150.6 (C-2), 159.7 (C-4), 164.8 (C-7′), 166.4 (C-3′’), ten aromatic or olefinic carbons, containing four vinyl carbons at *δ*_C_ 145.8 (C-6), 147.2 (C-7), 147.7 (C-9), 127.2 (C-10), one O-methyl at *δ*_C_ 53.5 (O-CH3), one O-methylene at *δ*_C_ 64.7 (O-CH2-). ^1^H and ^13^C NMR (Table 2) spectra analysis revealed that some signals of **3** was similar to that of compound **4** [9] and 2, 2-dimethyl-1, 3-dioxa-benzo[d]pentane-6-carboxylic acid [19]. With the aid of the ^1^H, ^1^H-COSY, HSQC, and HMBC correlations, the structure of **3** was established as shown (Figure 1 and Figure 2). The absolute configuration of **3** at C-2″ was also determined as 2″*R* by ECD calculations (Figure 3).

### 2.2. Biological Activity

**Figure 4:**
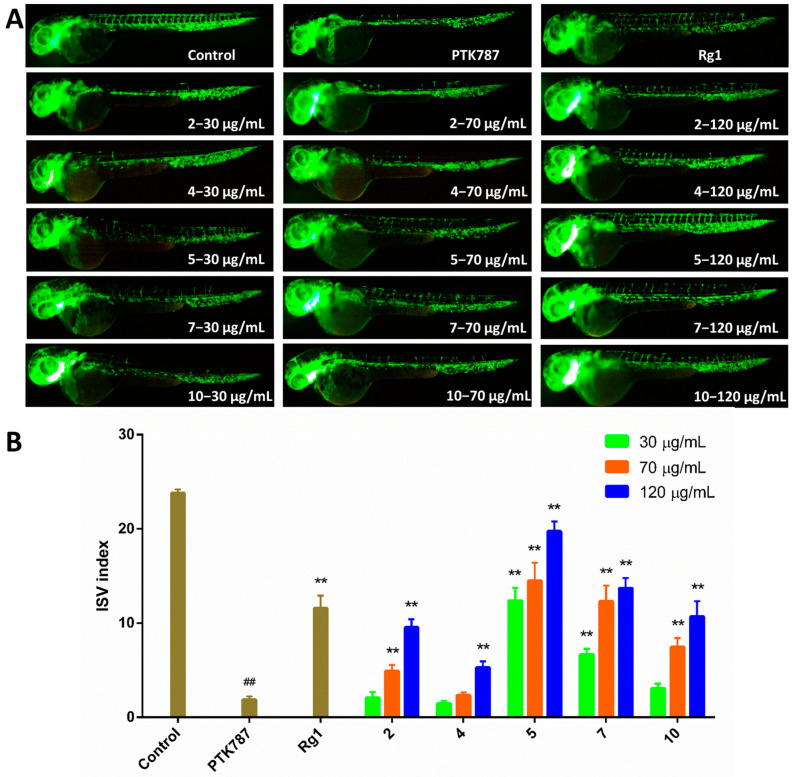
Results of pro-angiogenesis activities. (**A**) Typical images of intersomitic vessels (ISV) in transgenic fluorescent zebrafish (Tg (vegfr2: GFP)) treated with PTK787 and different concentrations (30, 70 and 120 μg/mL) of compounds **2**, **4, 5**, **7**, and **10**, using ginsenoside Rg1 (120 μg/mL) as a positive control. (**B**) Quantitative analysis of the ISV index (number of intact vessels * 1+number of defective vessels * 0.5) in zebrafish treated with compounds **2**, **4**, **5**, **7**, and **10**. Data represented as mean ± SEM. ^##^ *p* < 0.01 compared to the control group; ** *p* < 0.01 compared to the PTK787 group.

**Figure 5:**
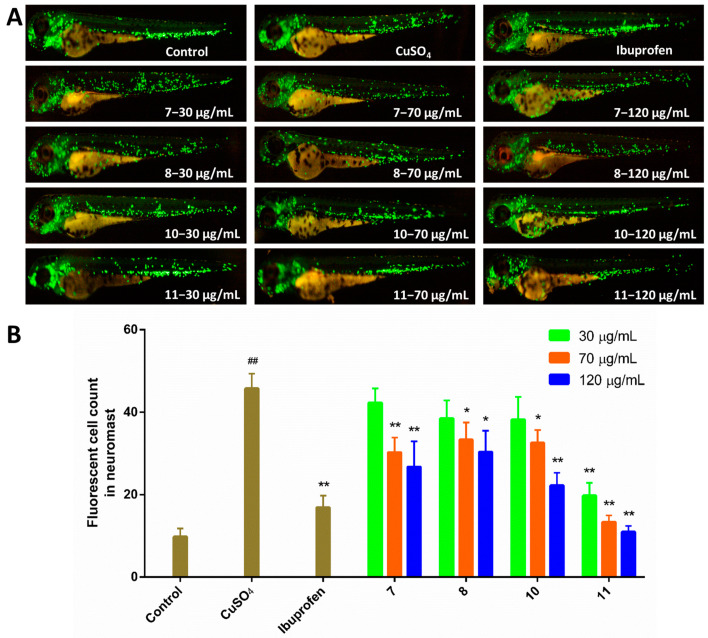
Results of anti-inflammatory activities. (**A**) Typical images on inflammatory sites in CuSO_4_-induced transgenic macrophages fluorescent of compounds **7**, **8**, **10**, and **11**, using ibuprofen (10 μM) as a positive control. (**B**) Quantitative analysis of the number of fluorescent macrophages. The data are represented as the mean ± SEM. ^##^ *p* < 0.01 compared to the control group; * *p* < 0.05 and ** *p* < 0.01 compared to the CuSO_4_ group.

In previous report, some alkaloids from marine-derived fungus showed pro-angiogenic activities in a zebrafish model [8]. We are also committed to find more marine natural products with angiogenesis related activity. In the present study, all isolated compounds were tested for the pro-angiogenic activities in a vatalanib (PTK787) induced vascular injury zebrafish model (Table S1). Compounds **5** and **7** (at concentrations of 30, 70 and 120 μg/mL) significantly promoted the angiogenesis, compounds **2** and **10** (70 and 120 μg/mL) also had effects, and compounds **4** (120 μg/mL) exhibited moderate effects (Figure 4). Compared to compound **7**, compounds **8** and **9** were inactive with respect to pro-angiogenesis, indicating that phenolic hydroxyl group is necessary for pro-angiogenic activity. All compounds were also evaluated for anti-inflammatory effects in CuSO_4_-induced zebrafish inflammation model (Table S1). Compound **11** (30, 70, and 120 μg/mL) displayed potent anti-inflammatory activity, and compounds **7**, **8**, and **10** (70, and 120 μg/mL) had moderate effects (Figure 5). Compound **7** showed better anti-inflammatory activity than **8**, while **9** was ineffective, suggesting the phenolic hydroxyl group and the epoxide oxygen are important in the anti-inflammatory activity. Meanwhile, compared to compound **11**, compounds **12**–**14** displayed no anti-inflammatory activity, indicating that both phenolic and alcohol-hydroxyl groups are necessary for anti-inflammatory activity. In addition, all compounds were tested for cytotoxicity against human liver carcinoma cells HepG2 by MTT method (Table S1) [20], and compound **6** exhibit cytotoxicity with an IC_50_ value of 30 µg/mL (Figure S1). The pro-angiogenic, anti-inflammatory activities in zebrafish and cytotoxicity against HepG2 cells of these compounds were reported here for the first time.

## 3. Materials and Methods

### 3.1. General Experimental Procedures

Optical rotations were measured on a JASCO P-2000 digital polarimeter (JASCO, Tokyo, Japan). UV spectra were performed on an Eppendorf BioSpectrometer Basic photometer. IR spectra were recorded on a JASCO FT/IR-4600 spectrometer in KBr discs. CD data were obtained on a JASCO J-810 spectropolarimeter. NMR spectra were collected using a JEOL JNM-ECP 600 spectrometer (JEOL, Tokyo, Japan). HRESIMS data were acquired on an Agilent 6210 ESI/TOF mass spectrometer (Agilent, Santa Clara, CA, USA). Analytical high performance liquid chromatography (HPLC) system (Waters, Milford, MA, USA) consisted of Waters e2695, UV Detector 2489, and software Empower using a C18 column (Diamonsil C18(2), 250 × 4.6 mm, 5 μM). Semipreparative HPLC was operated on the same system using a C18 column (Cosmosil 5C18-MS-II, 250 × 10 mm, 5 μM). Vacuum-liquid chromatography (VLC) used silica gel H (Qingdao Marine Chemical Factory, Qingdao, China). Thin layer chromatography (TLC) and column chromatography were performed on plates pre-coated with silica gel GF254 (10–40 μm) and Sephadex LH-20 (GE Healthcare Biosciences, Uppsala, Sweden), respectively.

### 3.2. Fungal Material

The fungus Y32-2 was isolated from the seawater sample collected from a depth of about 30 m in the Indian Ocean (88°59′51″ E, 2°59′54″ S) in 2013. It was identified as *Aspergillus austroafricanus* (GenBank access No. MK267449) by rDNA amplification and sequence analysis of the ITS region. The producing strain was prepared on potato dextrose agar medium stored at 4 °C.

### 3.3. Fermentation and Extraction

The fungus was cultured in 500 mL Erlenmeyer flasks with fermentation media containing 80 g of rice and 120 mL of sea water at 28 °C for 40 days. The whole fermented material was extracted exhaustively with EtOAc. Then the EtOAc extract was dried under reduced pressure to obtain residue (30.1 g).

### 3.4. Purification and Identification

The EtOAc extract was subjected to silica gel chromatography with a vacuum liquid chromatography (VLC) column, using a stepwise gradient solvent system of petroleum ether (PE)-CH_2_Cl_2_ (7:3, 3:7 and 0:1), then of CH_2_Cl_2_-MeOH (99:1, 49:1, 19:1, 9:1, 4:1, 1:1, and 0:1) to obtain thirteen primary fractions (Fr.1–Fr.13). Fr.6–Fr.11 were individually subjected to Sephadex LH-20 column (120 × 2 cm) chromatography with CH_2_Cl_2_-MeOH (1:1) as mobile phase, and then fractions were purified separately by semipreparative HPLC column (Cosmosil 5C18-MS-II, 250 × 10 mm, 5 μM) using different gradients of MeOH in H_2_O. Fr.6 (3.5 g) afforded **6** (70% MeOH-H_2_O, *v*/*v*; *t*_R_ = 23.5 min; 12.4 mg), **8** (60% MeOH-H_2_O, *v*/*v*; *t*_R_ = 20.5 min; 91.2 mg), **9** (60% MeOH-H_2_O, *v*/*v*; *t*_R_ = 21.9 min; 12.8 mg), **13** (65% MeOH-H_2_O, *v*/*v*; *t*_R_ = 25.9 min; 8.6 mg), **14** (60% MeOH-H_2_O, *v*/*v*; *t*_R_ = 24.8 min; 4.7 mg). Fr.7 (2.7 g) afforded **4** (40% MeOH-H_2_O, *v*/*v*; *t*_R_ = 18.5 min; 4.4 mg). Fr.8 (1.5 g) afforded **1** (70% MeOH-H_2_O, *v*/*v*; *t*_R_ = 10.4 min, 14.4min; 6.5 mg), **7** (60% MeOH-H_2_O, *v*/*v*; *t*_R_ = 16.9 min; 21.4 mg), **11** (60% MeOH-H_2_O, *v*/*v*; *t*_R_ = 22.8 min; 5.6 mg), **12** (65% MeOH-H_2_O, *v*/*v*; *t*_R_ = 26.0 min; 14.3 mg). Fr.9 (0.5 g) afforded **3** (65% MeOH-H_2_O, *v*/*v*; *t*_R_ = 28.5 min; 4.5 mg), 10 (60% MeOH-H_2_O, *v*/*v*; *t*_R_ = 22.5 min; 5.5 mg). Fr.10 (1.1 g) afforded **2** (60% MeOH-H_2_O, *v*/*v*; *t*_R_ = 15.0 min; 5.1 mg). Fr.11 (1.3 g) afforded **5** (50% MeOH-H_2_O, *v*/*v*; *t*_R_ = 14.5 min; 3.0 mg).

*Di-6-hydroxydeoxybrevianamide E* (**1**)*:* Yellow amorphous powder; ${\left[\alpha \right]}_{D}^{20}$ +24 (*c* 0.1, MeOH); UV (MeOH) λ_max_ 216, 299 nm; IR (KBr) ν_max_ 3460, 2973, 2925, 1667, 1440, 1306, 1192, 1108, 1001, 920, 809 cm^−1^; ^1^H and ^13^C NMR data, see Table 1; HRESIMS *m*/*z* 731.3559 [M − H]^−^ (calcd. for C_42_H_48_N_6_O_6_, 731.3557).

*Dinotoamide J* (**2**)*:* Yellow amorphous powder; ${\left[\alpha \right]}_{D}^{20}$ +22 (*c* 0.1, MeOH); UV (MeOH) λ_max_ 210, 226 and 295 nm; IR (KBr) ν_max_ 3447, 1646, 1442, 1186, 1105, 618 cm^−1^; ^1^H and ^13^C NMR data, see Table 1; HRESIMS *m*/*z* 763.3440 [M − H]^−^ (calcd. for C_42_H_48_N_6_O_8_, 763.3456).

*Asperpteridinate A:* Yellow amorphous powder; ${\left[\alpha \right]}_{D}^{20}$ +63 (*c* 0.1, MeOH); UV (MeOH) λ_max_ 218, 239, 300, 334 nm; ^1^H and ^13^C NMR data, see Table 2; IR (KBr) ν_max_ 3465, 1633, 1263, 1192, 1105, 615 cm^−1^; HRESIMS *m*/*z* 465.1018 [M + Na]^+^ (calcd. for C_20_H_18_N_4_O_8_, 465.1023).

### 3.5. ECD Computational Calculation

The conformational analyses were carried out by random searching in the Sybyl-X 2.0 using the MMFF94S force field with an energy cutoff of 5.0 kcal/mol [21]. Subsequently, the conformers were re-optimized using DFT at the PBE0-D3/def2-SVP level in MeOH using the polarizable conductor calculation model (SMD) by the GAUSSIAN 09 program [22]. The energies, oscillator strengths, and rotational strengths (velocity) of the first 30 electronic excitations were calculated using the TDDFT methodology at the CAM-B3LYP-D3/def2-SVP level in MeOH. The ECD spectra were simulated by the overlapping Gaussian function (half the bandwidth at 1/e peak height, sigma = 0.30 for all) [23]. To get the final spectra, the simulated spectra of the conformers were averaged according to the Boltzmann distribution theory and their relative Gibbs free energy (∆G).

### 3.6. Bioassay Protocols

#### 3.6.1. Cell Culture and Cytotoxicity Assay

According to previous report [24], The HepG2 cells were cultured with DMEM medium, pH 7.0, supplemented with 10% FBS and 1% antibiotics (10,000 IU mL^−1^ of penicillin and 10 mg mL^−1^ of streptomycin), and the culture flasks were incubated under a humidified atmosphere of 37 °C and 5% CO_2_. The cytotoxic activities of all compounds against HepG2 cells in vitro were determined by modified MTT assays as described previously [21]. Cells were seeded into a 96-well plate at a density 5 × 10^4^ per well. After overnight incubation, the cells were treated with the chemicals for 24 h, and 10 μL MTT (5 mg/mL) was added to each well at 37 °C for 4 h, then 100 μL lysis buffer was added for the cell lysis. The OD value of each sample was detected at 560 nm using a microplate reader. The experiments were carried out in triplicate.

#### 3.6.2. Zebrafish Maintenance

The zebrafish (*Danio rerio*) strains used in this assay were the AB wild-type, Tg (vegfr2-GFP) and Tg (zlyz-EGFP) transgenic lines [25,26]. They were maintained at 28.0 °C ± 0.5 °C in an automatic circulating tank system with light-dark cycle (14 h:10 h). The healthy adult zebrafish were placed in a breeding tank in the evening, and mated in the next morning. The fertilized eggs were collected, disinfected with methylene blue solution, and then raised in clean culture water including 5.0 mM NaCl, 0.17 mM KCl, 0.4 mM CaCl_2_, and 0.16 mM MgSO_4_ in a light-operated incubator.

#### 3.6.3. Pro-Angiogenesis Assay

Vascular insufficiency in zebrafish was modeled by VEGFR tyrosine kinase inhibitor PTK787 to evaluate the effects of compounds on pro-angiogenesis according to previous report [8,26]. The healthy zebrafish larvae were separated into 24-well plates (ten embryos per well) in a 2 mL final volume of culture water at 24 h post fertilization (hpf). 0.2 μg/mL PTK787 was co-treated with each test compound (30, 70, 120 μg/mL) as test group. The control group was fresh culture water, the model group was 0.2 μg/mL PTK787, the positive drug group was 0.2 μg/mL PTK787 and 120 μg/mL ginsenoside Rg1. After 24 h incubation in a light-operated incubator at 28.0 °C ± 0.5 °C, the number of intersegmental blood vessels (ISVs) were captured by a fluorescent microscope (Olympus, SZX2-ILLTQ, Tokyo, Japan). Intact and defective vessels were counted separately and ISVs index was defined as follows: ISV index = number of intact vessels × 1 + number of defective vessels × 0.5 [27]. The zebrafish larvae without PTK787 in test group was used to evaluate the effects of compounds on anti-angiogenesis under the same conditions above described. All treatments were performed in triplicate.

#### 3.6.4. Anti-Inflammatory Assay

The zebrafish inflammation model was induced by CuSO_4_ to evaluate the effects of compounds on anti-inflammation [28]. In total, 72 hpf zebrafish larvae were distributed into 24-well plates (ten embryos per well) in a 2 mL final volume of culture water, and treated with different concentrations of each test compound (30, 70, 120 μg/mL) for 2 h as test group. Then CuSO_4_ was added and incubated for 1 h. The control group was fresh culture water, the model group was 20 μM CuSO_4_ and the positive drug group was 20 μM CuSO_4_ and 10 μM ibuprofen. After 4 h incubation in a light-operated incubator at 28.0 °C ± 0.5 °C, the number of macrophages were imaged by a fluorescent microscope (Olympus, SZX2-ILLTQ, Tokyo, Japan). All treatments were performed in triplicate.

#### 3.6.5. Statistical Analysis

Statistical analysis were processed by GraphPad Prism 6.0 software. All the experimental data were shown as mean ± SEM. The comparison between groups was performed by student’s test. * *p* < 0.05 was considered as significant difference. ** *p* < 0.01 was a very significant difference.

## 4. Conclusions

To summarize, two new indole alkaloid dimers di-6-hydroxydeoxybrevianamide E (**1**), dinotoamide J (**2**) and one new pteridine alkaloid asperpteridinate A (**3**), together with eleven known compounds (**4**–**14**) were isolated from the marine-derived fungus *Aspergillus austroafricanus* Y32-2. Their structures including the absolute configurations were elucidated by various spectroscopic methods and ECD calculations. Among them, both di-6-hydroxydeoxybrevianamide E (**1**) and dinotoamide J (**2**) are homologous dimers that represent the novel examples of prenylated indole alkaloids. Asperpteridinate A is the first new alkaloid composed of pteridine and 1, 3-benzodioxole structures. All compounds were evaluated for pro-angiogenic, anti-inflammatory activities in the zebrafish models and cytotoxicity against HepG2 cells. Compounds **2**, **4**, **5**, **7**, and **10** exhibited pro-angiogenic activity, and compounds **7**, **8**, **10**, and **11** displayed anti-inflammatory activity in a dose-dependent manner, and compound **6** showed significant cytotoxicity against HepG2 cells with an IC_50_ value of 30 µg/mL. The results suggested that these compounds could be promising candidates for further pharmacologic and biosynthetic research.

## Figures and Tables

**Table 1:** 400 MHz ^1^H and 150 MHz ^13^C NMR data of compounds **1** and **2** in DMSO-*d*_6_.

No.	1		2	
	*δ*_C_ Type	*δ*_H_ (Mult., *J* in Hz)	*δ*_C_ Type	*δ*_H_ (Mult., *J* in Hz)
2,2′	139.1, 139.2 C	―	180.1 C	―
3,3′	104.75, 104.82 C	―	55.9 C	―
4,4′	117.6, 117.8 CH	7.35, 7.37 (each H, d, 8.4)	126.0 CH	7.11 (2H, d, 8.2)
5,5′	109.9, 110.0 CH	6.80, 6.81 (each H, d, 8.4)	106.9 CH	6.39 (2H, d, 8.2)
6,6′	150.0, 150.1 C	―	155.7 C	―
7,7′	104.0, 104.1 C	―	104.0 C	―
8,8′	134.4, 134.5 C	―	143.4 C	―
9,9′	122.6, 122.7 C	―	118.4 C	―
10,10′	25.3, 25.4 CH_2_	2.94, 3.52 (each 2H, m)	30.3 CH_2_	2.03 (2H, dd, 14.7, 5.2) 2.80 (2H, dd, 14.7, 4.7)
11,11′	55.0, 55.3 CH	4.33, 4.38 (each H, dd, 9.2, 4.2)	52.6 CH	3.41 (2H, dd, 5.2, 4.7)
12,12′	165.6 C	―	165.6 C	―
14,14′	44.8 CH_2_	3.37, 3.47 (each 2H, m)	45.1 CH_2_	3.23, 3.35 (each 2H, m)
15,15′	22.2 CH_2_	1.75−1.91 (4H, m)	22.3 CH_2_	1.67−1.79 (4H, m)
16,16′	27.6 CH_2_	1.85, 2.12 (each 2H, m)	27.1 CH_2_	1.77, 1.99 (each 2H, m)
17,17′	58.5 CH	4.22 (2H, t-like, 7.2)	58.3 CH	4.02 (2H, t-like, 7.6)
18,18′	169.3, 169.4 C	―	169.8 C	―
20,20′	111.29, 111.33 CH_2_	5.01 (2H, br d, 10.7) 5.06 (2H, br d, 17.5)	112.6 CH_2_	4.94 (2H, br d, 17.5) 5.00 (2H, br d, 10.9)
21,21′	146.4, 146.5 CH	6.15, 6.17 (each H, dd, 17.5, 10.7)	143.9 CH	6.15 (2H, dd, 17.5, 10.9)
22,22′	38.6, 38.7 C	―	42.2 C	―
23,23′	27.5, 27.6 CH_3_	1.42 (6H, s)	21.1 CH_3_	0.98 (6H, s)
24,24′	27.5, 27.6 CH_3_	1.42 (6H, s)	22.6 CH_3_	0.96 (6H, s)
1,1′-NH	―	8.45, 8.58 (each H, s)	―	9.31 (2H, s)
19,19′-NH	―	6.21, 6.30 (each H, s)	―	7.57 (2H, s)
6,6′-OH	―	9.09 (2H, br s)	―	9.28 (2H, br s)

**Table 2:** 400 MHz ^1^H and 150 MHz ^13^C NMR data of compound **3** in DMSO-*d*_6_.

No.	*δ*_C_, Type	*δ*_H_, (Mult., *J* Hz)
2	150.6 C	―
4	159.7 C	―
6	145.8 C	―
7	147.2 CH	8.94 (1H, s)
9	147.7 C	―
10	127.2 C	―
11	64.7 CH_2_	5.49 (2H, s)
1′	123.6 C	―
2′	109.2 CH	7.49 (1H, d, 1.4)
3′	147.0 C	―
4′	150.9 C	―
5′	108.8 CH	7.10 (1H, d, 8.3)
6′	125.9 CH	7.65 (1H, dd, 8.3, 1.4)
7′	164.8 C	―
1″	21.7 CH_3_	1.90 (3H, s)
2″	112.6 C	―
3″	166.4 C	―
N1-Me	29.2 CH_3_	3.53 (3H, s)
N3-Me	28.7 CH_3_	3.31 (3H, s)
3″-OMe	53.5 CH_3_	3.74 (3H, s)

## Supplementary Materials

The following are available online at https://www.mdpi.com/1660-3397/19/2/98/s1, HRESIMS, 1D and 2D NMR spectra of all new compounds **1**–**3**, biological activities of all isolated compounds and dose–response curves of compound **6**, the atom coordinates and energies of new compounds **1**–**3**.
