# Supplementary material for: New Prenylated Indole Homodimeric and Pteridine Alkaloids from the Marine-Derived Fungus Aspergillus austroafricanus Y32-2
Source: Mar Drugs. 2021 Feb 9;19(2):98. doi: 10.3390/md19020098 (PMC7916005; doi:10.3390/md19020098)
Supplement: Supplementary file 1 [file marinedrugs-19-00098-s001.pdf]

Supplementary Materials

New Prenylated Indole Homodimeric and Pteridine Alkaloids from the Marine-Derived Fungus *Aspergillus austroafricanus* Y32-2

Peihai Li [1,2,3†], Mengqi Zhang [1,2,3†], Haonan Li [1], Rongchun Wang [1,3], Hairong Hou [1,3], Xiaobin Li [1,3,*], Kechun Liu [1,3,*] and Hao Chen [4,*]

[1] Engineering Research Center of Zebrafish Models for Human Diseases and Drug Screening of Shandong Province, Shandong Provincial Engineering Laboratory for Biological Testing Technology, Biology Institute, Qilu University of Technology (Shandong Academy of Sciences), Jinan 250103, China; liph@sdas.org (P.L.); mengqi@sdas.org (M.Z.); 17862958363@163.com (H.L.); wangrc@sdas.org (R.W.); houhr@sdas.org (H.H.)

[2] State Key Laboratory of Biobased Material and Green Papermaking, Qilu University of Technology, Shandong Academy of Sciences, Jinan, 250353, China

[3] Key Laboratory for Biosensor of Shandong Province, Biology Institute, Qilu University of Technology (Shandong Academy of Sciences), Jinan 250103, China

[4] Key Laboratory of Marine Bioactive Substances, First Institute of Oceanography, Ministry of Natural Resources, Qingdao, 266061, China

* Correspondence: lixb@sdas.org (X.L.), hliukch@sdas.org (K.L.), hchen@fio.org.cn (H.C.); Tel./Fax: +86-531-82605352 (X.L.), +86-531-82605331 (K.L.), +86-532-88963855 (H.C.)

† These authors contributed equally to this work.

List of Supplementary Materials

Table S1. All biological activities of isolated compounds……………….…………………………S3
Figure S1. Dose–response curves of compound **6**…………………………………………………S3
Figure S2. HRESIMS data of compound **1**…………………………………………………………S4
Figure S3. ^{1}H-NMR spectrum of compound **1** in DMSO-d_6……..…………………………………S5
Figure S4. ^{13}C-NMR spectrum of compound **1** in DMSO-d_6……..………………………………S6
Figure S5. HSQC spectrum of compound **1** in DMSO-d_6………..………………………………S7
Figure S6. ^{1}H-^{1}H COSY spectrum of compound **1** in DMSO-d_6…………………………………S8
Figure S7. HMBC spectrum of compound **1** in DMSO-d_6…………..……………………………S9
Figure S8. NOESY spectrum of compound **1** in DMSO-d_6……..…………………………………S10
Figure S9. HRESIMS data of compound **2**………………..…………..………………………………S11
Figure S10. ^{1}H-NMR spectrum of compound **2** in DMSO-d_6………………………………………S12
Figure S11. ^{13}C-NMR spectrum of compound **2** in DMSO-d_6…………..………………………S13
Figure S12. HSQC spectrum of compound **2** in DMSO-d_6…………..…………………………S14
Figure S13. ^{1}H-^{1}H COSY spectrum of compound **2** in DMSO-d_6……………..………………S15
Figure S14. HMBC spectrum of compound **2** in DMSO-d_6…………...…………………………S16
Figure S15. NOESY spectrum of compound **2** in DMSO-d_6…………..…………………………S17
Figure S16. HRESIMS data of compound **3**……………………………………………………………S18
Figure S17. ^{1}H-NMR spectrum of compound **3** in DMSO-d_6…………..…………………………S19
Figure S18. ^{13}C-NMR spectrum of compound **3** in DMSO-d_6……………………………………S20
Figure S19. HSQC spectrum of compound **3** in DMSO-d_6…………………..…………………S21
Figure S20. ^{1}H-^{1}H COSY spectrum of compound **3** in DMSO-d_6………………..………………S22
Figure S21. HMBC spectrum of compound **3** in DMSO-d_6………………….………...……S23
Table S2. Cartesian coordinates for the low-energy reoptimized random research conformers of compound **1**…………………………………………………………………………….………………S24
Table S3. The atom energies of the low-energy conformers of compound **1**……………………S35
Table S4. Cartesian coordinates for the low-energy reoptimized random research conformers of compound **2**…………………………………………………………………………….………………S36
Table S5. The atom energies of the low-energy conformers of compound **2**……………………S44
Table S6. Cartesian coordinates for the low-energy reoptimized random research conformers of compound **3**…………………………………………………………………………….………………S44
Table S7. The atom energies of the low-energy conformers of compound **3**……………………S62

Table S1. All biological activities of isolated compounds

Compounds	Bioactivities (μg/mL)		
	Pro-angiogenesis	Anti-inflammatory	cytotoxicity against HepG2 (IC_{50})
**1**	-	-	>150
**2**	70, 120	-	>150
**3**	-	-	>150
**4**	120	-	>150
**5**	30, 70, 120	-	>150
**6**	-	-	30
**7**	30, 70, 120	70, 120	>150
**8**	-	70, 120	>150
**9**	-	-	>150
**10**	70, 120	70, 120	>150
**11**	-	30, 70, 120	>150
**12**	-	-	>150
**13**	-	-	>150
**14**	-	-	>150

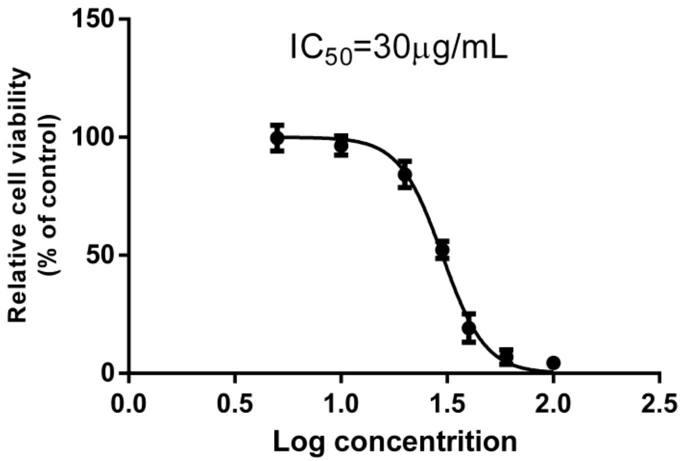

Figure S1. Dose–response curve of compound **6**

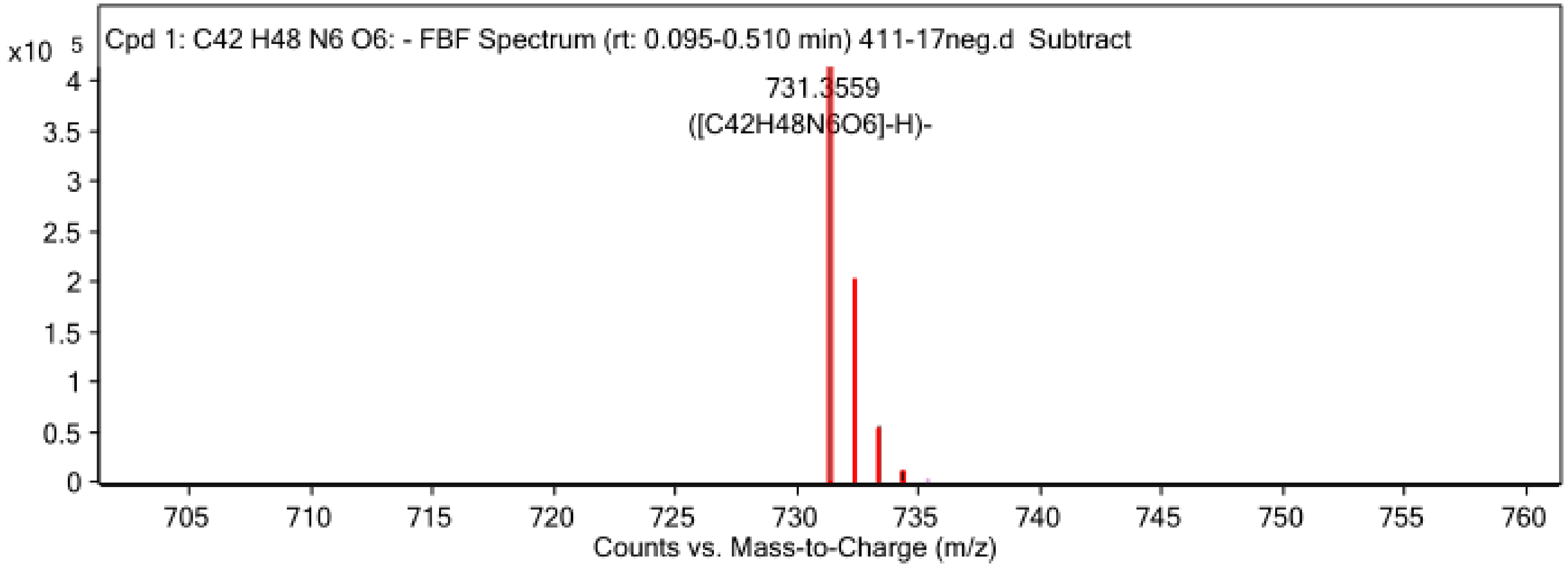

MS Spectrum Peak List

m/z	z	Abund	Formula	Ion
731.3559	1	413059.88	C42H48N6O6	(M-H)-
732.3593	1	201177.38	C42H48N6O6	(M-H)-
733.3622	1	55169.09	C42H48N6O6	(M-H)-
734.3649	1	11227.97	C42H48N6O6	(M-H)-

--- End Of Report ---

Figure S2. HRESIMS data of compound **1**.

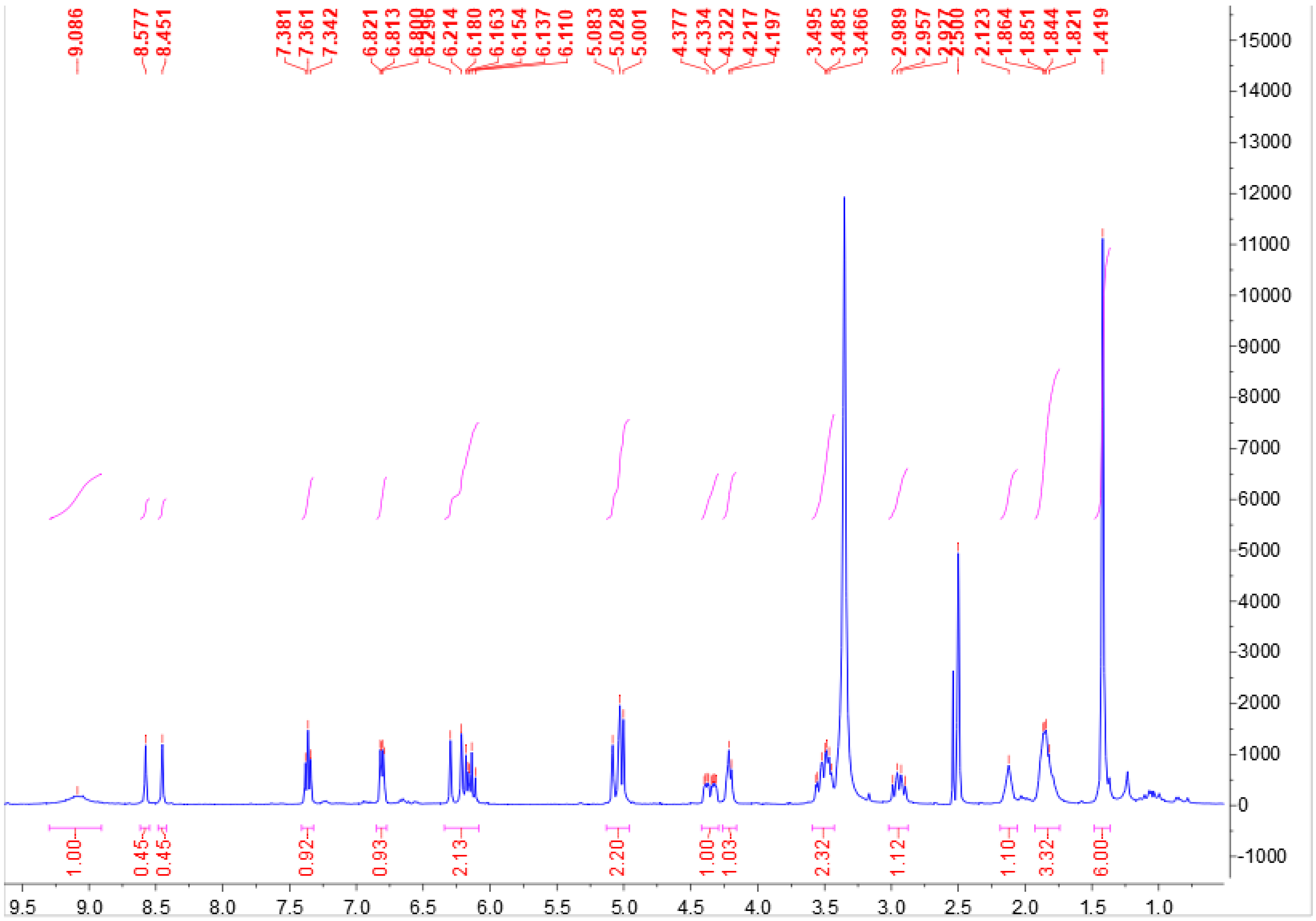

Figure S3. ^{1}H-NMR (400MHz) spectrum of compound **1** in DMSO-d_6.

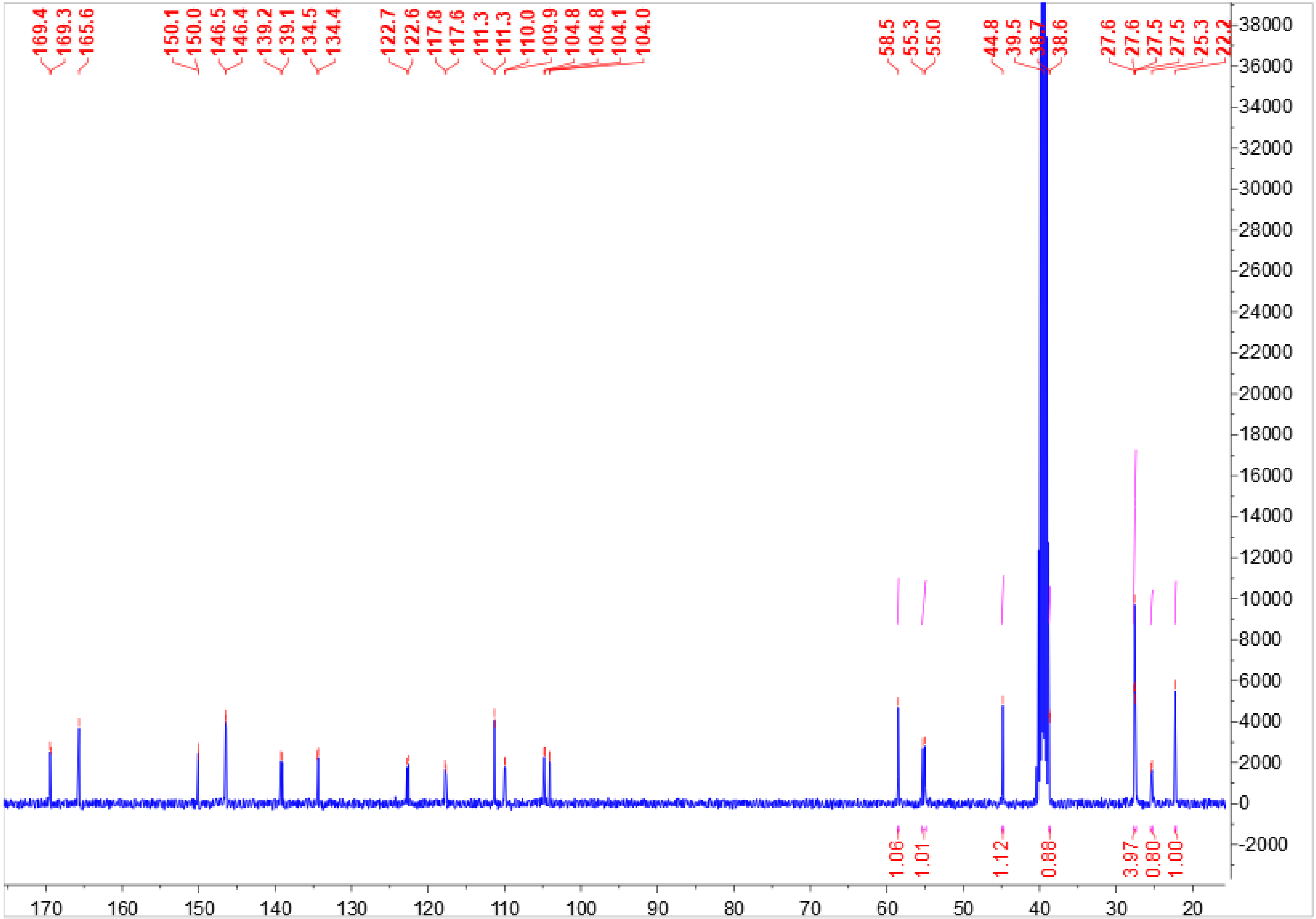

Figure S4. ^{13}C-NMR (150MHz) spectrum of compound **1** in DMSO-d_6.

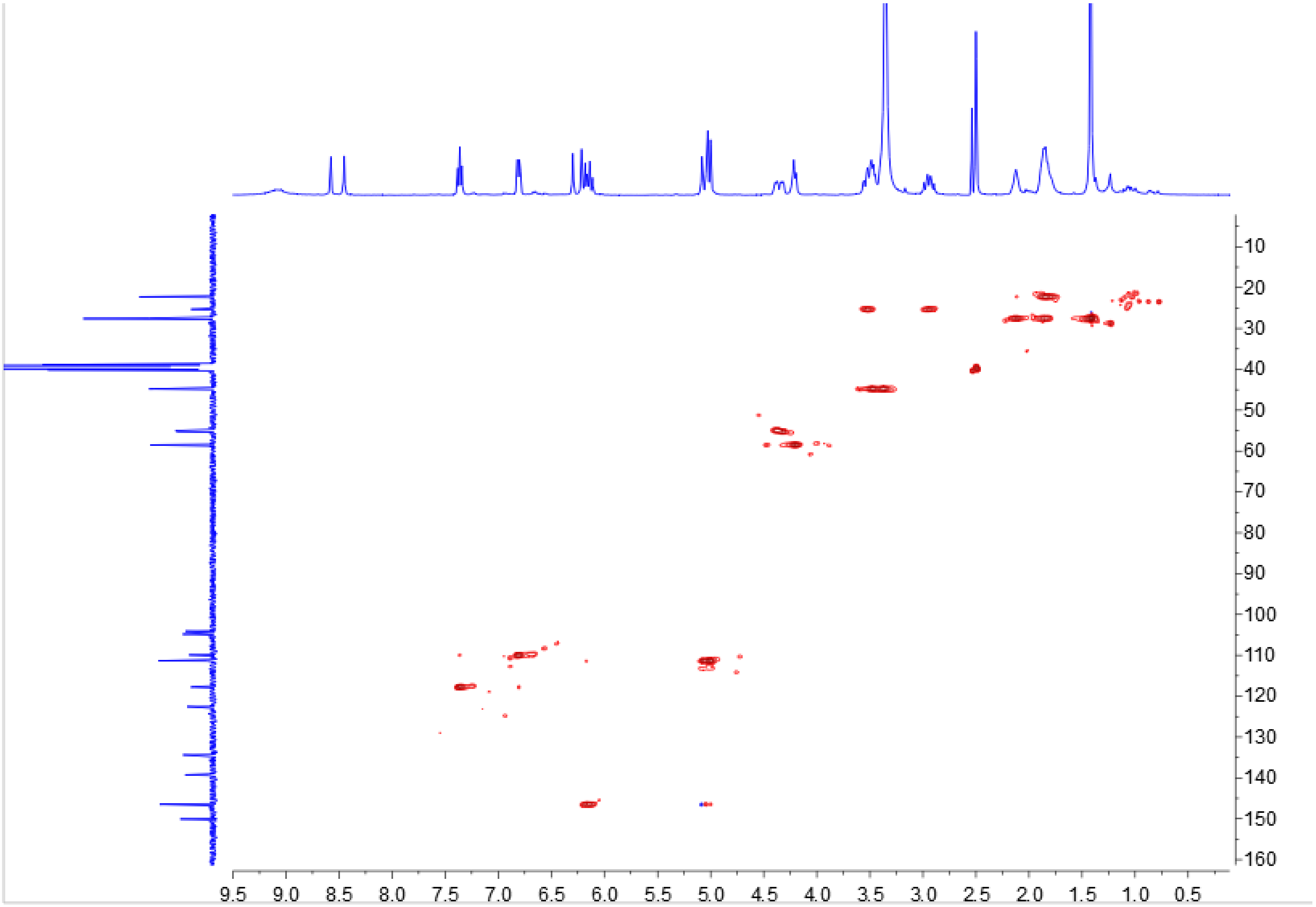

Figure S5. HSQC (400MHz) spectrum of compound **1** in DMSO-d_6.

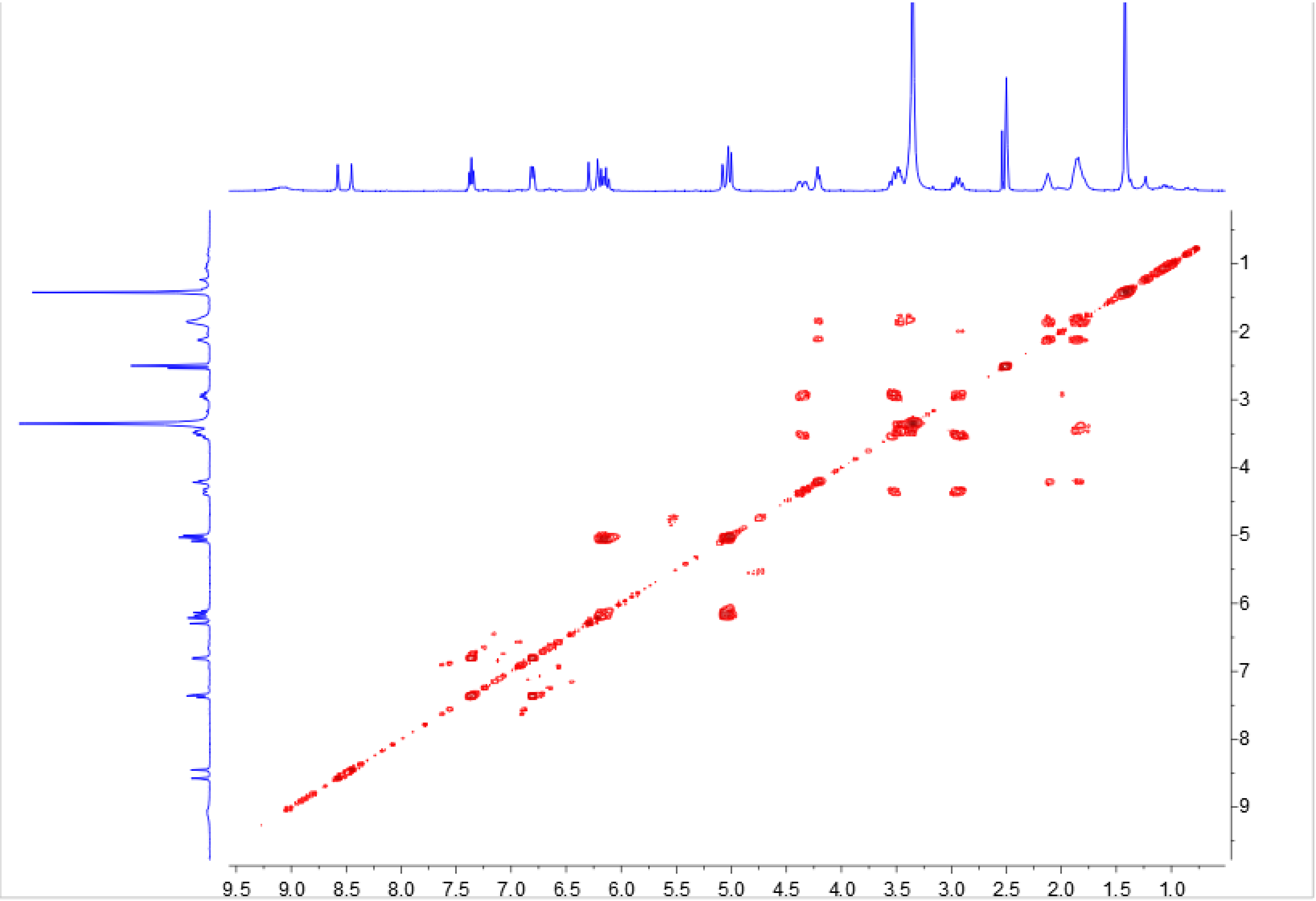

Figure S6. ^{1}H-^{1}H COSY (400MHz) spectrum of compound **1** in DMSO-d_6.

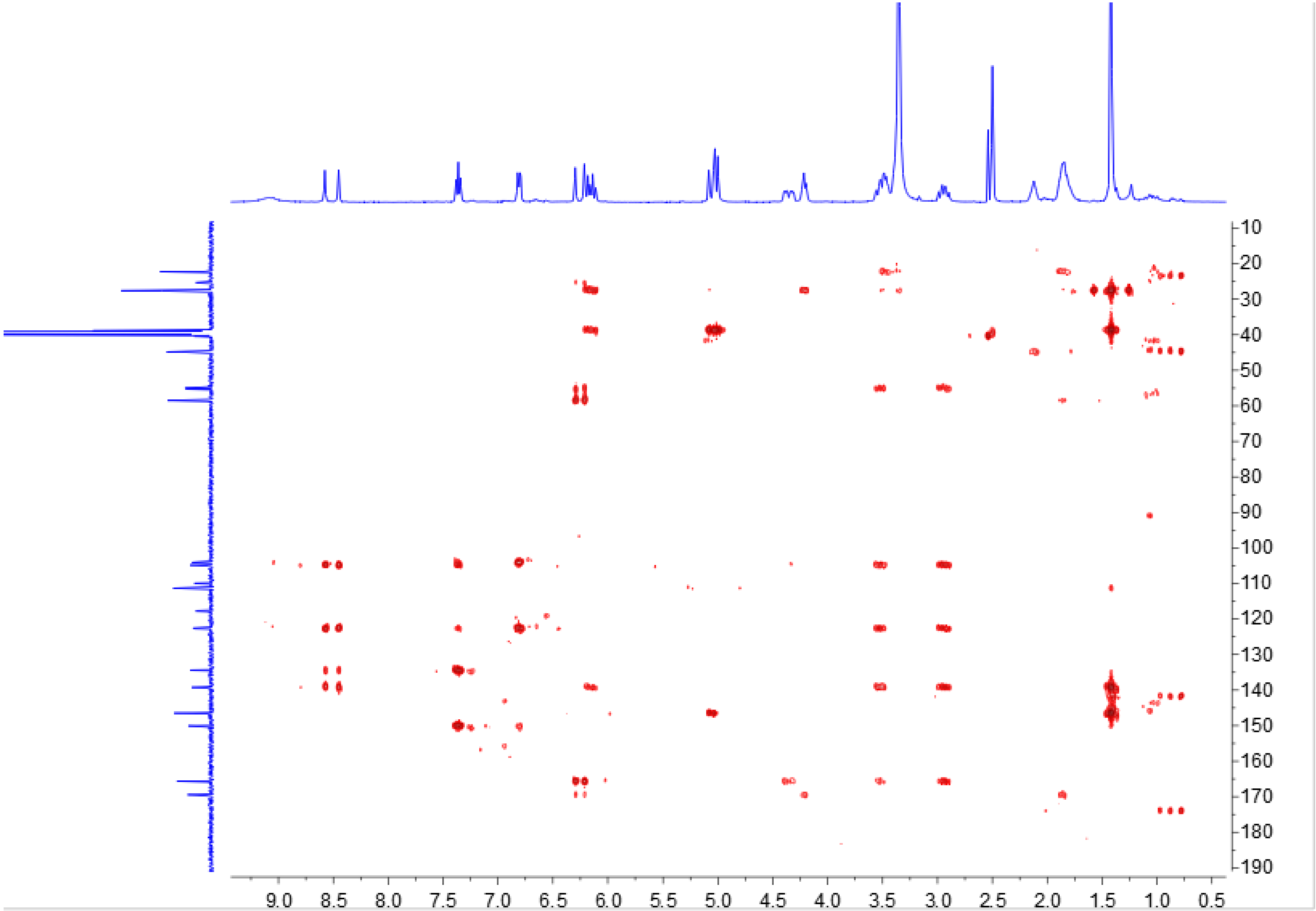

Figure S7. HMBC (400MHz) spectrum of compound **1** in DMSO-d_6.

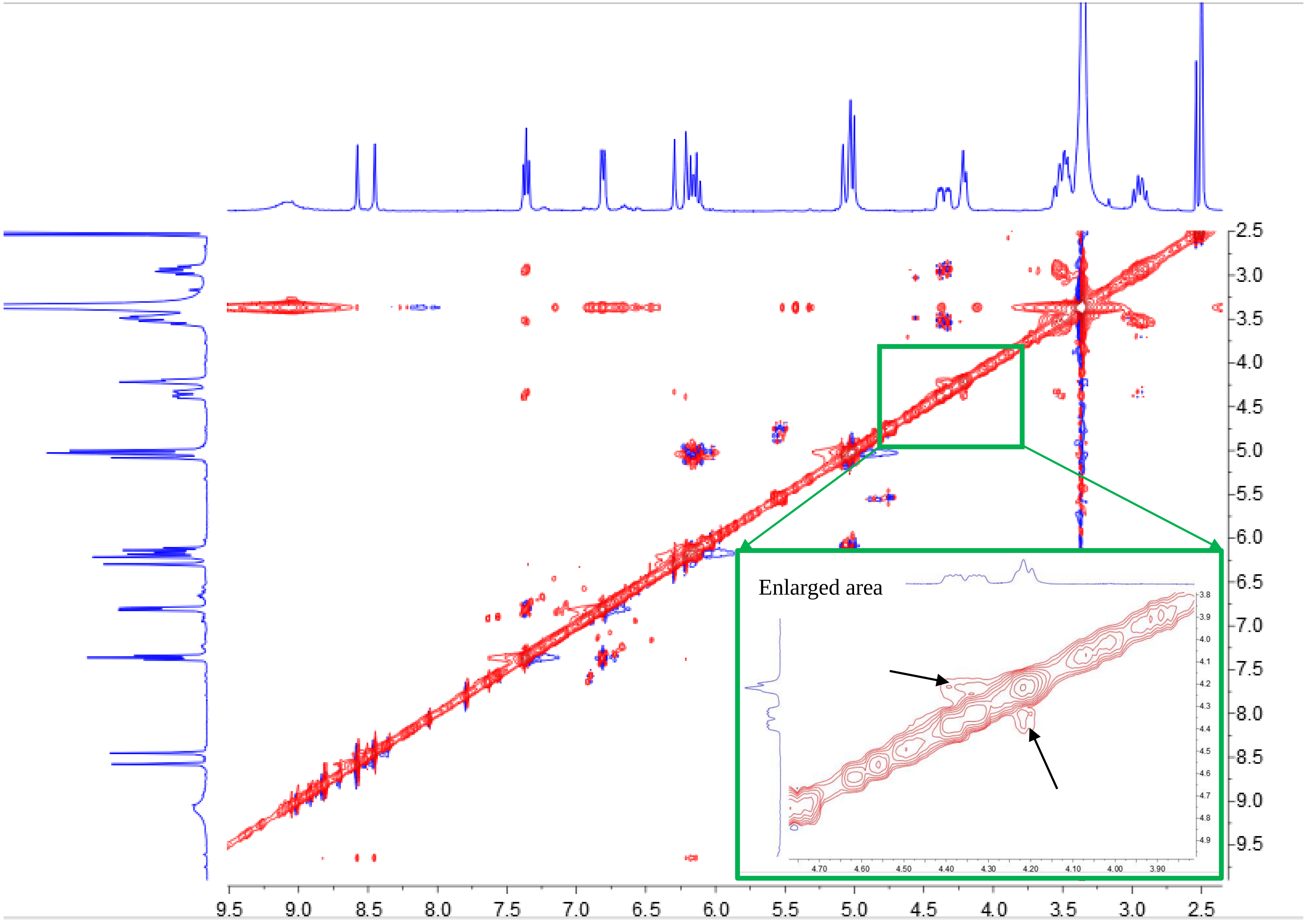

Figure S8. NOESY (400MHz) spectrum of compound **1** in DMSO-d_6. The NOESY correlations of H-11 and H-17 were indicated by black arrows in the enlarged area.

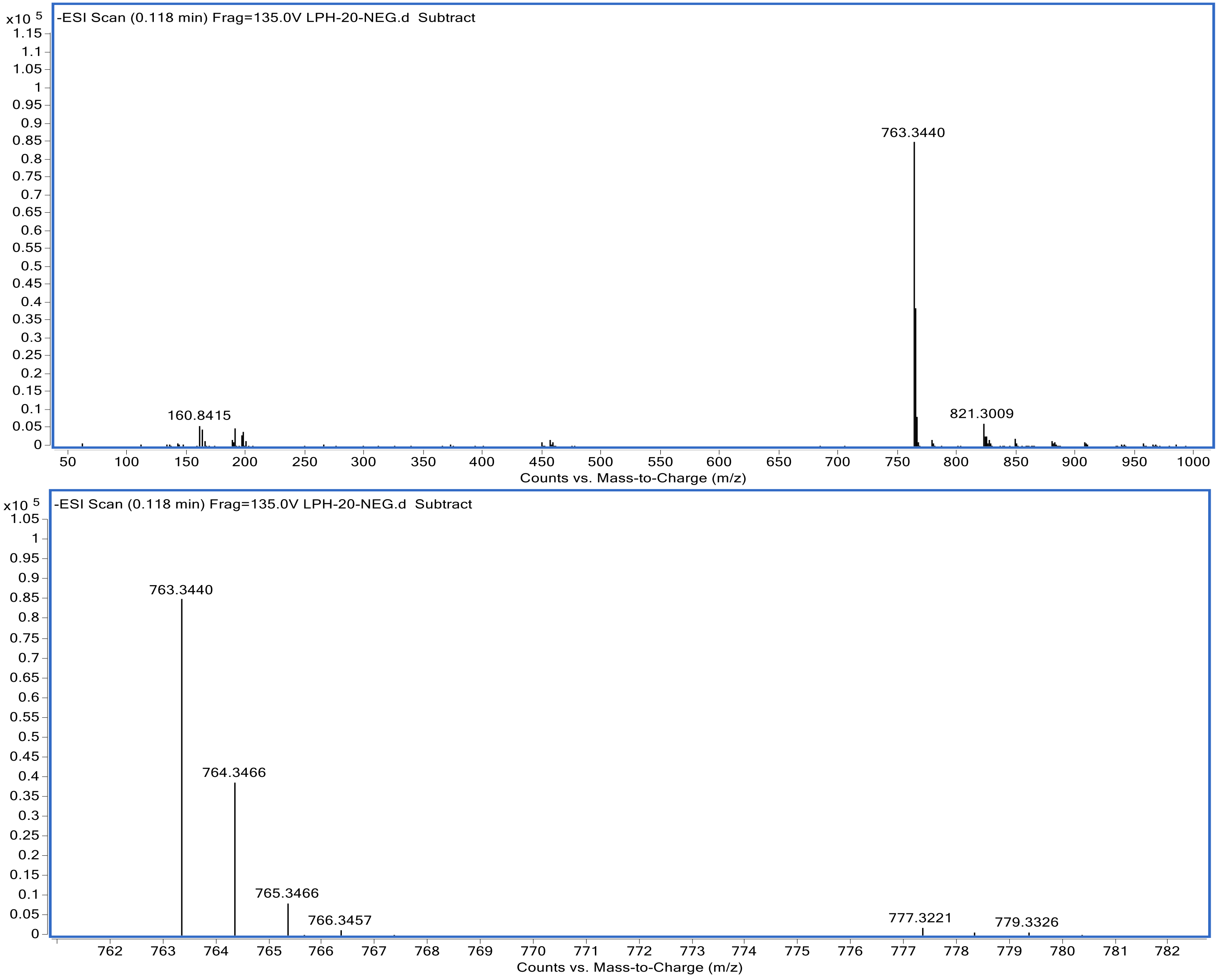

Figure S9. HRESIMS data of compound **2**

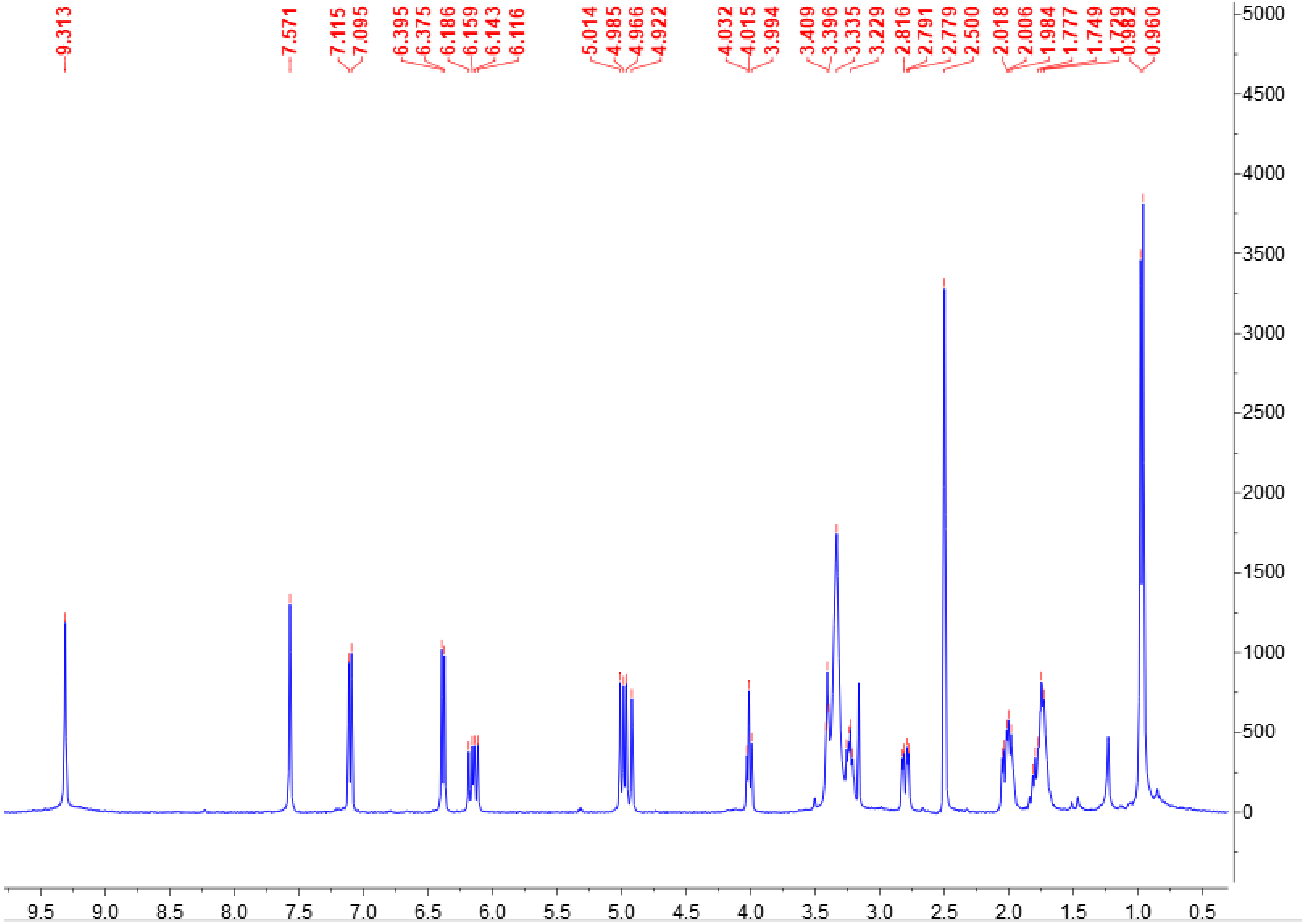

Figure S10. ^{1}H-NMR (400MHz) spectrum of compound **2** in DMSO-d_6.

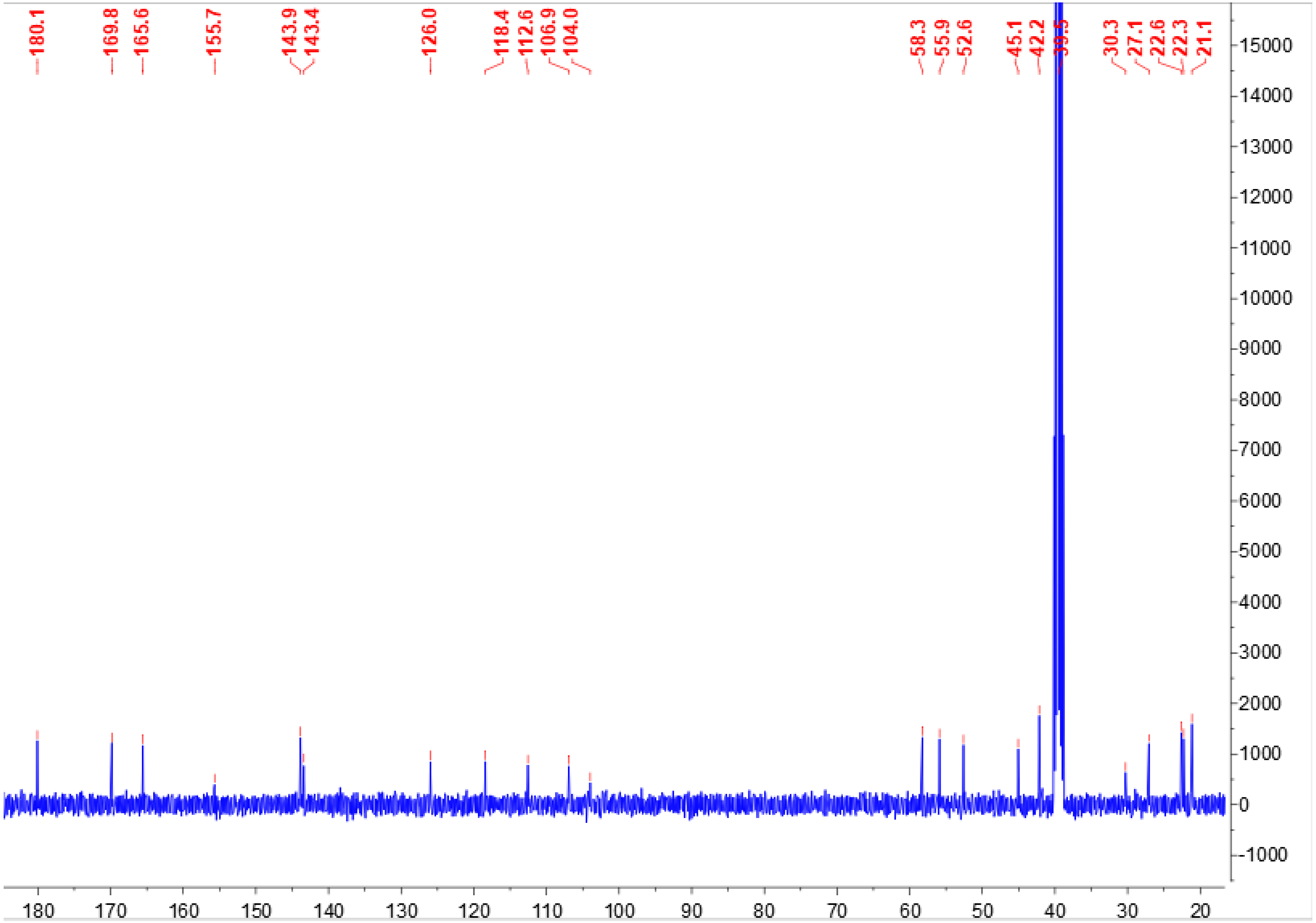

Figure S11. ^{13}C-NMR (150MHz) spectrum of compound **2** in DMSO-d_6.

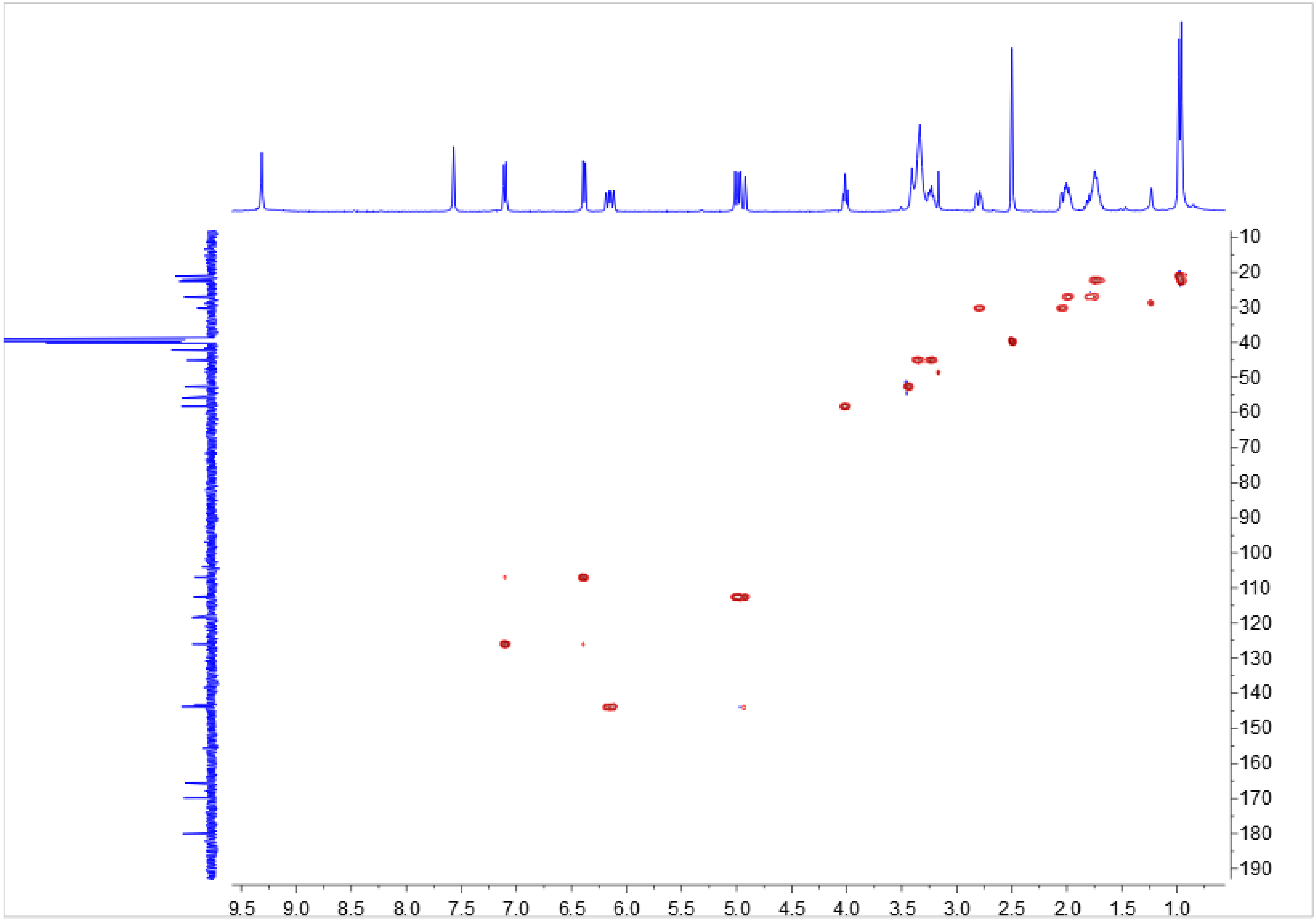

Figure S12. HSQC (400MHz) spectrum of compound **2** in DMSO-d_6.

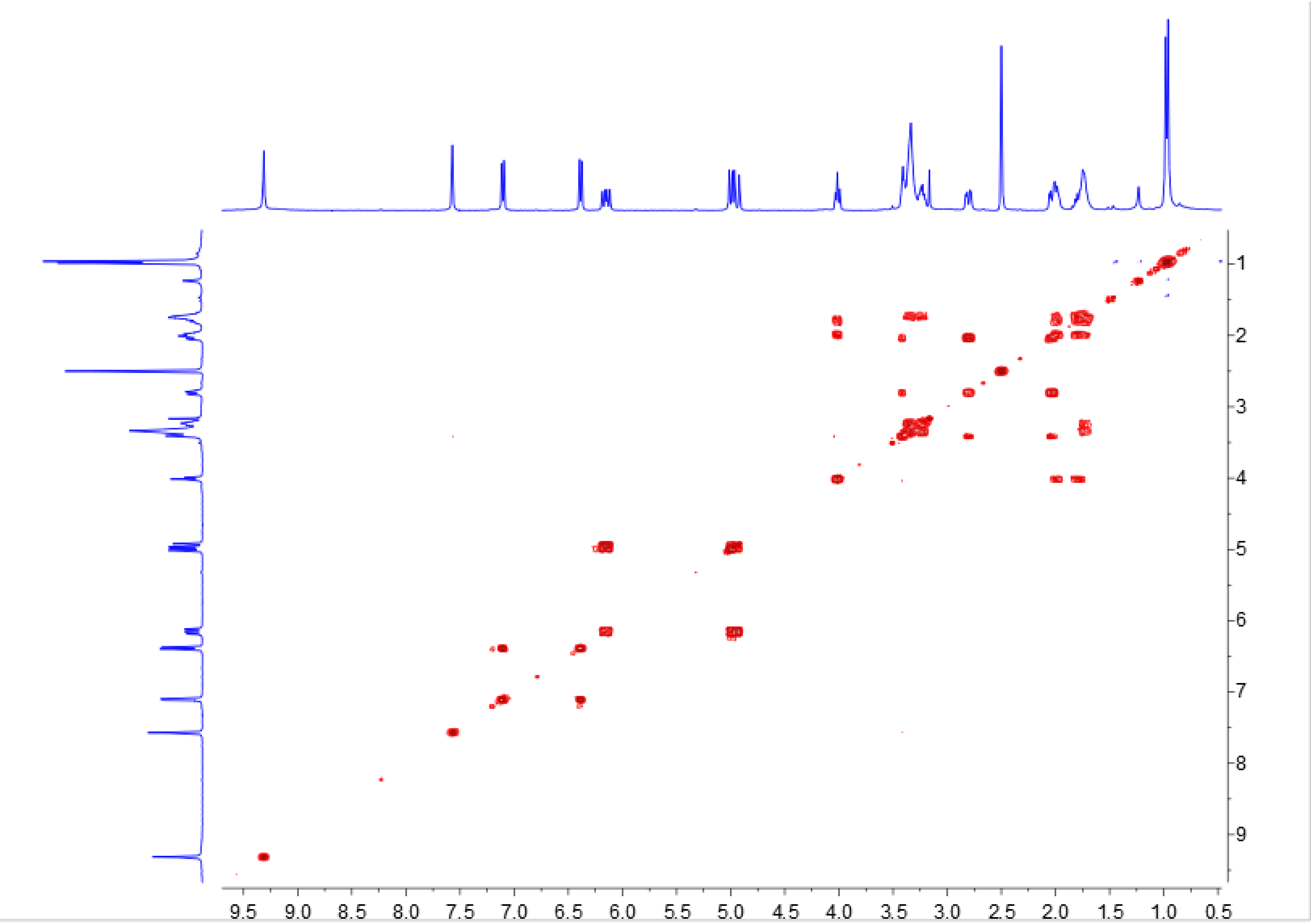

Figure S13. ^{1}H-^{1}H COSY (400MHz) spectrum of compound **2** in DMSO-d_6.

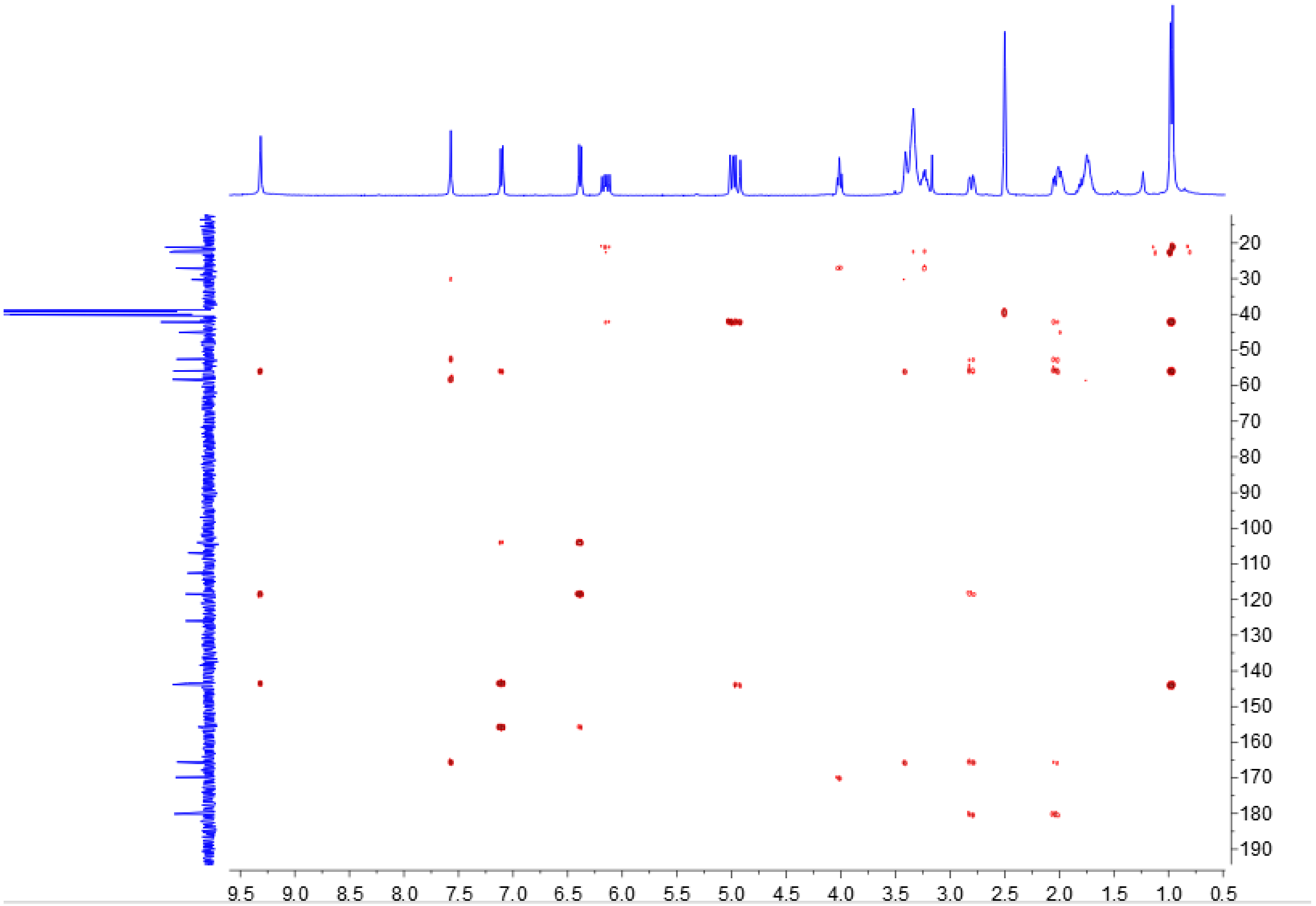

Figure S14. HMBC (400MHz) spectrum of compound **2** in DMSO-d_6.

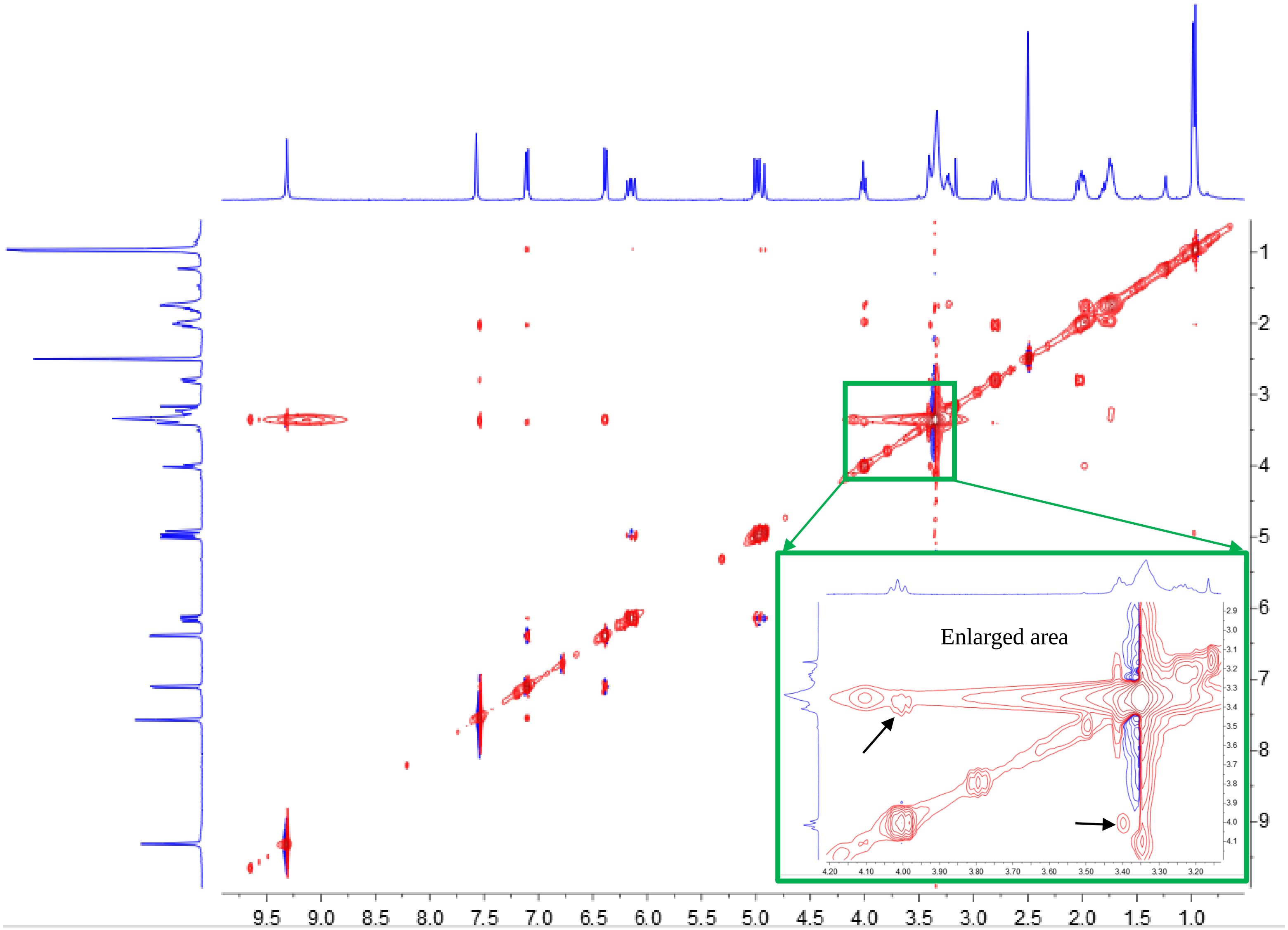

Figure S15. NOESY (400MHz) spectrum of compound **2** in DMSO-d_6. The NOESY correlations of H-11 and H-17 were indicated by black arrows in the enlarged area.

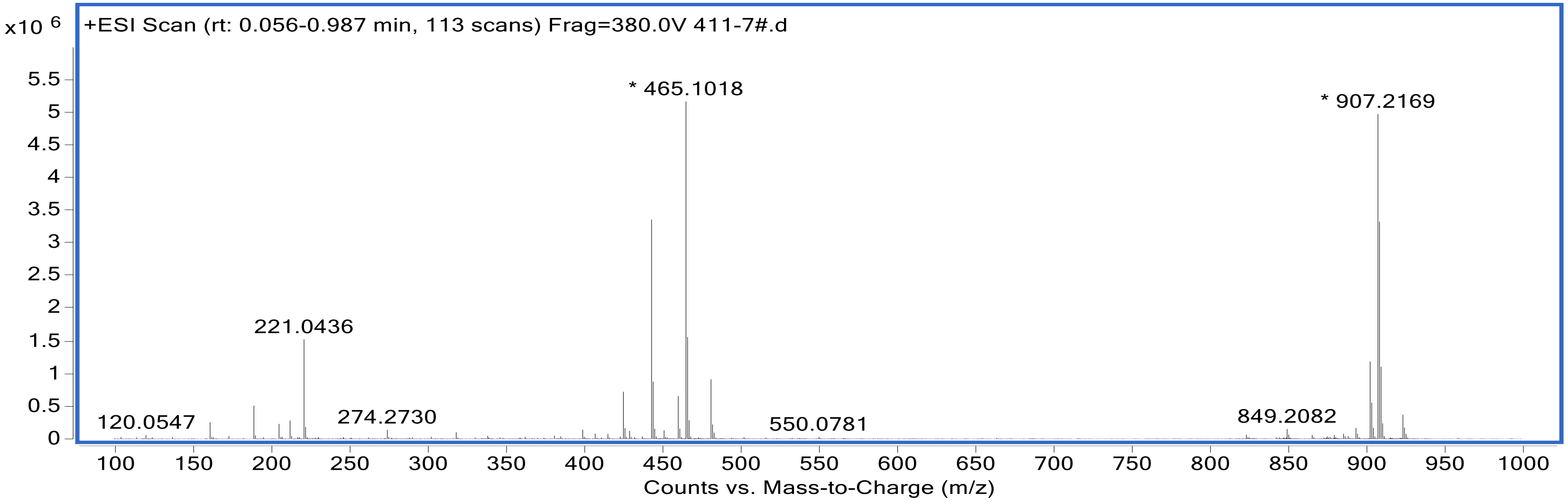

Figure S16. HRESIMS data of compound **3**

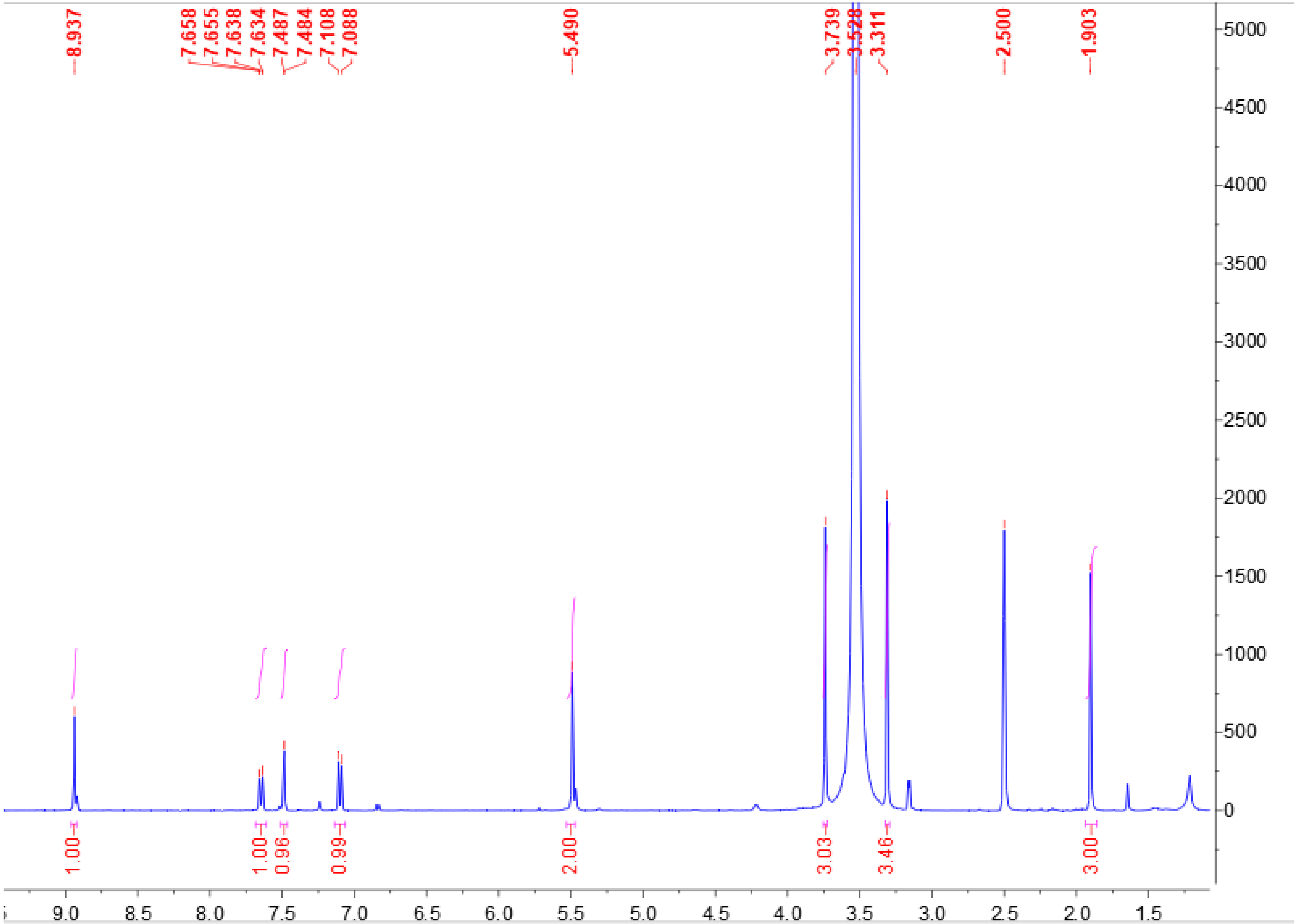

Figure S17. ^{1}H-NMR (400MHz) spectrum of compound **3** in DMSO-d_6.

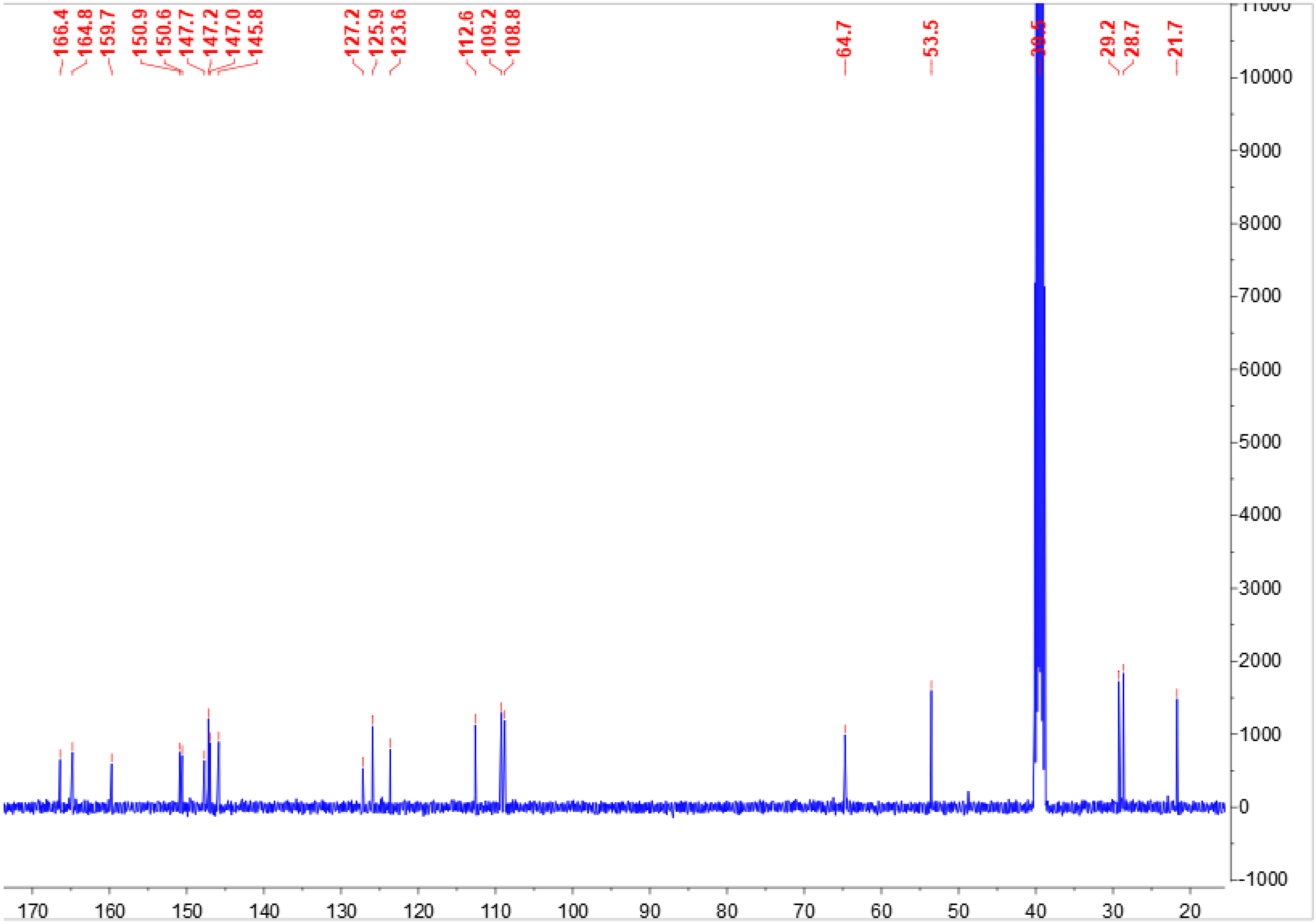

Figure S18. ^{13}C-NMR (150MHz) spectrum of compound **3** in DMSO-d_6.

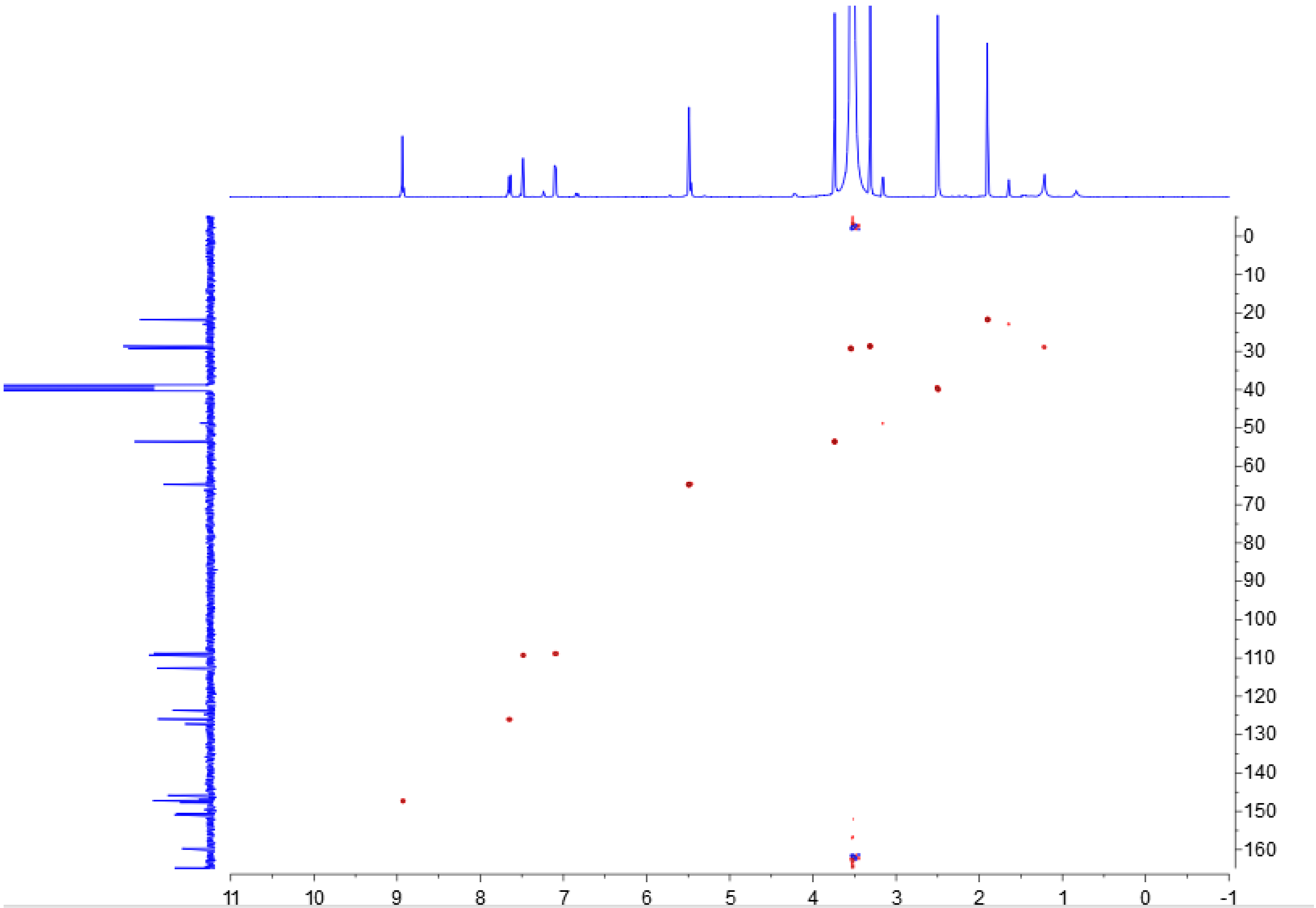

Figure S19. HSQC (400MHz) spectrum of compound **3** in DMSO-d_6.

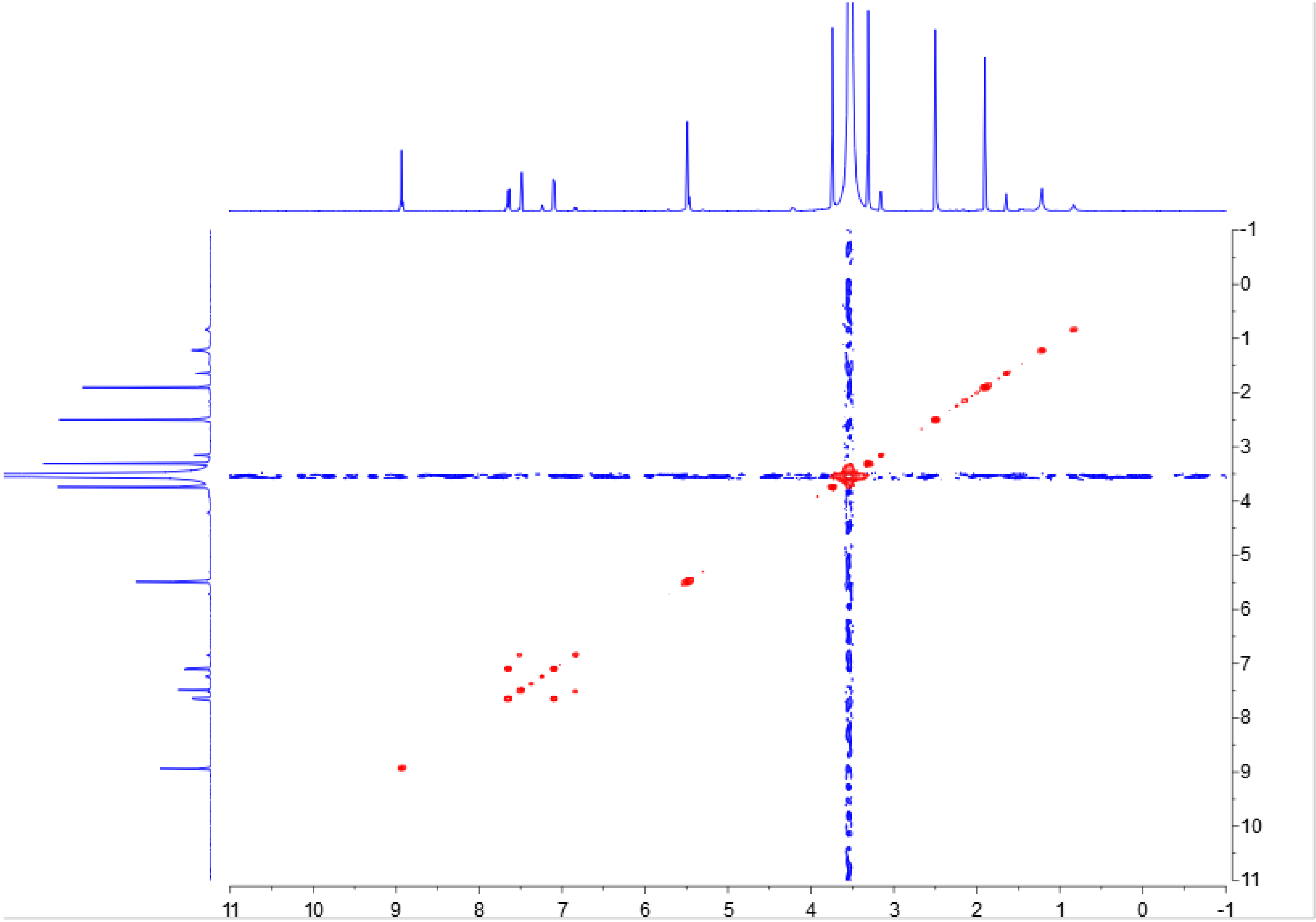

Figure S20. ^{1}H-^{1}H COSY (400MHz) spectrum of compound **3** in DMSO-d_6.

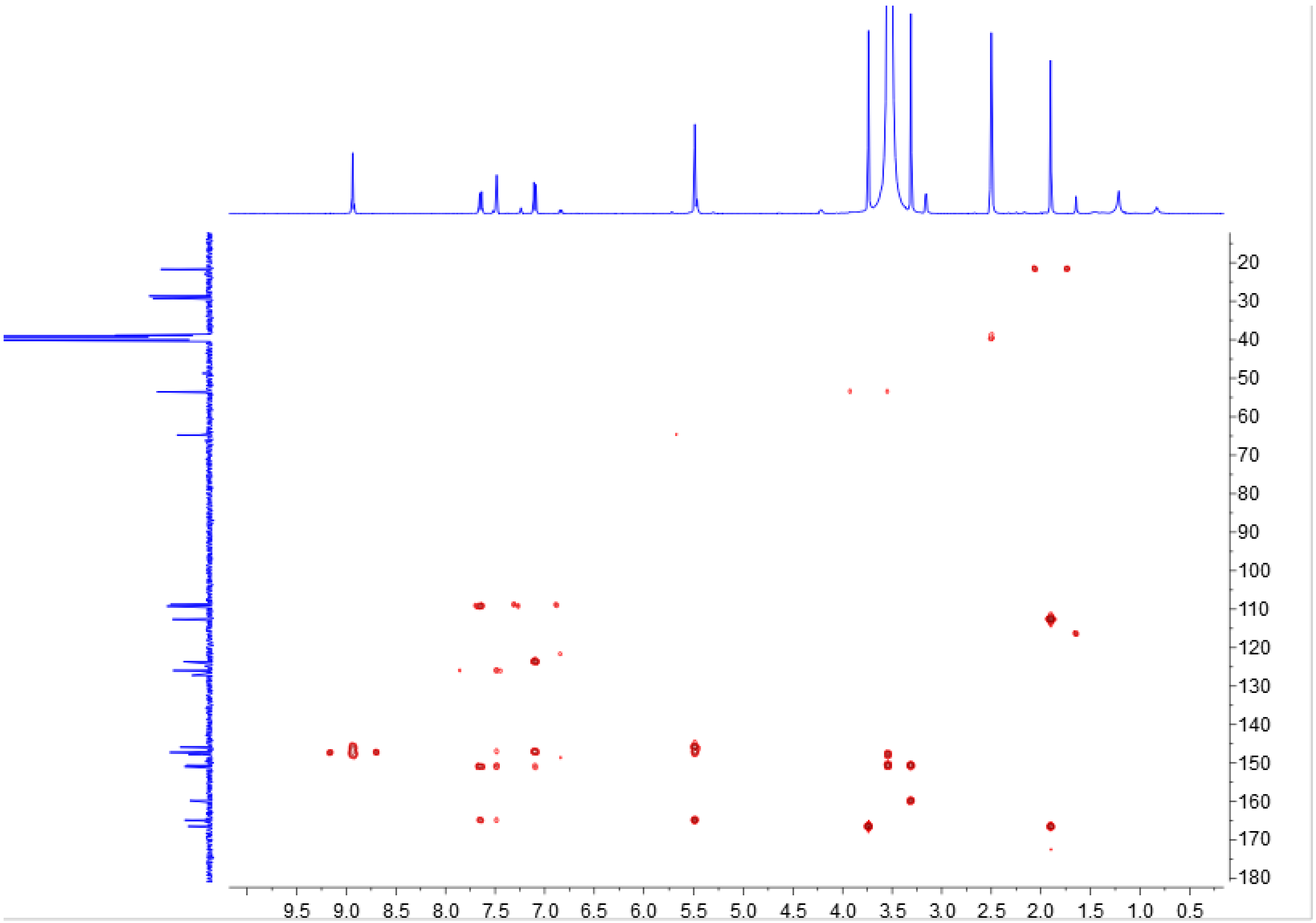

Figure S21. HMBC (400MHz) spectrum of compound **3** in DMSO-d_6.

Table S2. Cartesian coordinates for the low-energy reoptimized random research conformers of compound **1** at PBE0-D3/def2-SVP level of theory in methanol.

1_tddft_		Standard Orientation (Ångstroms)			
Center number	Atomic number	Atomic Type	X	Y	Z
0	6	0	-3.971659	5.001589	-4.928552
1	6	0	-2.562483	2.763244	-5.024116
2	6	0	-2.244555	1.238525	-2.88973
3	6	0	-3.60612	1.976663	-0.74059
4	6	0	-5.127973	4.171291	-0.63029
5	6	0	-5.216738	5.733186	-2.755218
6	7	0	-3.827081	0.808554	1.549416
7	6	0	-5.454488	2.132863	3.124941
8	6	0	-6.311218	4.23465	1.822717
9	6	0	-5.880523	0.992344	5.720776
10	7	0	-11.797681	3.191132	2.077738
11	6	0	-10.922251	5.721989	1.508938
12	6	0	-8.217132	6.173438	2.564608
13	6	0	-12.053577	1.271648	0.456935
14	6	0	-12.023475	1.89659	-2.302352
15	7	0	-11.530177	4.533464	-2.913704
16	6	0	-11.059034	6.38204	-1.269409
17	8	0	-12.490312	-0.924589	1.146112
18	8	0	-10.659627	8.599979	-1.927474
19	6	0	-10.138839	0.50456	-3.972473
20	6	0	-10.23853	2.054204	-6.417011
21	6	0	-11.029992	4.741683	-5.628877

22	8	0	-1.427475	2.188954	-7.291502
23	1	0	-12.16358	7.055328	2.468487
24	1	0	-13.931206	1.445168	-2.962467
25	6	0	-3.299161	0.417534	6.818816
26	6	0	-2.385671	-1.838298	7.416754
27	6	0	-7.16384	2.796005	7.581828
28	6	0	-7.494537	-1.394924	5.463962
29	6	0	4.934368	0.242011	6.625641
30	6	0	4.255604	4.162949	4.141641
31	6	0	10.147687	1.211153	5.025768
32	6	0	8.228644	1.88616	3.563706
33	1	0	14.331178	-2.610539	-3.344456
34	1	0	10.12125	-1.477233	0.4822
35	8	0	-2.519195	-2.898296	-6.270211
36	6	0	15.792067	-7.082573	-0.279757
37	6	0	17.208271	-6.954608	-2.784483
38	6	0	15.168665	-6.340911	-4.7246
39	8	0	11.992952	-5.819256	3.25128
40	8	0	10.354369	-4.27775	-6.562926
41	6	0	11.820608	-5.033559	1.048362
42	7	0	13.574994	-5.471888	-0.70238
43	6	0	13.48957	-4.502745	-3.292947
44	6	0	10.83264	-4.216216	-4.27152
45	6	0	7.231989	-3.987577	1.76421
46	6	0	9.586302	-3.448127	0.179293
47	7	0	9.074461	-3.841332	-2.4979
48	6	0	5.446771	1.516228	4.082265

49	6	0	5.004069	-2.409649	1.053322
50	6	0	4.317846	-0.069063	1.984331
51	7	0	2.163664	0.714785	0.719153
52	6	0	2.890005	-5.121665	-2.497141
53	6	0	3.195735	-3.062683	-0.866888
54	6	0	1.450677	-1.039355	-1.036919
55	6	0	-0.517574	-0.9401	-2.803493
56	6	0	-0.665066	-2.995691	-4.443917
57	6	0	0.980961	-5.062204	-4.279067
58	1	0	-4.084783	6.141652	-6.620591
59	1	0	-6.292639	7.466825	-2.743106
60	1	0	-2.889973	-0.754169	2.089715
61	1	0	-11.938801	2.73755	3.925713
62	1	0	-7.636713	8.039591	1.915203
63	1	0	-8.391825	6.313562	4.599051
64	1	0	-10.655407	-1.461722	-4.24824
65	1	0	-8.271053	0.579361	-3.117447
66	1	0	-11.630091	1.260944	-7.704534
67	1	0	-8.424714	2.066146	-7.37801
68	1	0	-9.563412	6.141684	-5.926649
69	1	0	-12.720242	5.366898	-6.621949
70	1	0	-1.450648	0.359937	-7.500384
71	1	0	-2.140218	2.08083	7.140713
72	1	0	-0.527106	-2.035587	8.244168
73	1	0	-3.431338	-3.573068	7.145674
74	1	0	-9.11174	3.193744	7.066165
75	1	0	-6.151494	4.576948	7.750102

76	1	0	-7.204909	1.919802	9.442877
77	1	0	-7.800695	-2.263507	7.30446
78	1	0	-9.328626	-0.945383	4.658911
79	1	0	-6.617764	-2.794175	4.237691
80	1	0	5.740289	-1.642361	6.739564
81	1	0	5.698108	1.36312	8.173337
82	1	0	2.908723	0.068751	6.914386
83	1	0	5.16203	5.286598	5.604899
84	1	0	2.248222	4.084577	4.596265
85	1	0	4.488222	5.158126	2.355265
86	1	0	12.075033	1.607544	4.465793
87	1	0	9.906295	0.224608	6.799352
88	1	0	8.623307	2.848864	1.794177
89	1	0	-2.339651	-4.330454	-7.389151
90	1	0	15.160193	-8.992049	0.163615
91	1	0	16.893751	-6.390923	1.311863
92	1	0	18.603094	-5.442097	-2.730056
93	1	0	18.191395	-8.709652	-3.195286
94	1	0	14.09099	-8.026447	-5.2135
95	1	0	15.90828	-5.521117	-6.453146
96	1	0	7.759107	-3.685373	3.719719
97	1	0	6.765141	-5.986271	1.586838
98	1	0	7.317954	-3.409202	-3.111778
99	1	0	1.35066	2.417439	0.921889
100	1	0	4.130899	-6.743937	-2.405922
101	1	0	0.735518	-6.627536	-5.572767
5_tddft_		Standard Orientation (Ångstroms)			

Center number	Atomic number	Atomic Type	X	Y	Z
0	6	0	-3.992226	5.231552	-4.571074
1	6	0	-2.498397	3.061909	-4.832434
2	6	0	-2.109642	1.395582	-2.815875
3	6	0	-3.463272	1.945506	-0.605211
4	6	0	-5.07509	4.057932	-0.337539
5	6	0	-5.255796	5.75541	-2.349282
6	7	0	-3.598258	0.62381	1.608575
7	6	0	-5.262501	1.770846	3.285595
8	6	0	-6.235012	3.901657	2.121444
9	6	0	-5.457215	0.607599	5.899105
10	7	0	-11.60009	2.41111	2.374389
11	6	0	-10.933622	5.025849	1.914761
12	6	0	-8.265447	5.64656	2.989853
13	6	0	-11.545279	0.525107	0.692924
14	6	0	-11.615254	1.250408	-2.041364
15	7	0	-11.425787	3.950163	-2.550167
16	6	0	-11.131876	5.774261	-0.8367
17	8	0	-11.664957	-1.731652	1.308518
18	8	0	-10.951194	8.043221	-1.412315
19	6	0	-9.623585	0.139436	-3.793074
20	6	0	-9.952689	1.755941	-6.172353
21	6	0	-10.96528	4.324155	-5.255751
22	8	0	-1.361288	2.696787	-7.142057
23	1	0	-12.275935	6.213174	2.927038
24	1	0	-13.473885	0.616743	-2.695334

25	6	0	-7.350365	1.942921	7.55447
26	6	0	-6.880109	3.311066	9.599927
27	6	0	-6.356315	-2.155342	5.677768
28	6	0	-2.820007	0.630775	7.101762
29	6	0	4.849846	-0.572606	6.722574
30	6	0	4.289269	3.590669	4.616572
31	6	0	10.111858	0.545615	5.412349
32	6	0	8.247609	1.324253	3.930842
33	1	0	14.212138	-2.039687	-2.610045
34	1	0	10.318272	-1.890206	0.741264
35	8	0	-2.317766	-2.404857	-6.568164
36	6	0	15.551805	-7.576636	-1.242707
37	6	0	15.9749	-7.767662	-4.074543
38	6	0	15.652104	-5.052794	-4.989868
39	8	0	11.691381	-7.027605	2.527666
40	8	0	10.648191	-3.284064	-6.646532
41	6	0	11.732199	-5.633671	0.644845
42	7	0	13.580069	-5.652358	-1.071205
43	6	0	13.648662	-3.925144	-3.242757
44	6	0	11.029947	-3.603828	-4.361347
45	6	0	7.222631	-4.359347	1.527192
46	6	0	9.643702	-3.730312	0.088726
47	7	0	9.190024	-3.614607	-2.628259
48	6	0	5.447808	0.943175	4.332885
49	6	0	5.055319	-2.65827	0.919333
50	6	0	4.367879	-0.411413	2.05565
51	7	0	2.259922	0.522026	0.812829

52	6	0	3.020474	-4.984978	-2.930986
53	6	0	3.295412	-3.09416	-1.104602
54	6	0	1.574006	-1.044024	-1.123734
55	6	0	-0.360669	-0.76528	-2.910909
56	6	0	-0.485468	-2.663628	-4.735296
57	6	0	1.150949	-4.74271	-4.737685
58	1	0	-4.162112	6.478802	-6.180692
59	1	0	-6.417742	7.426998	-2.211616
60	1	0	-2.702274	-1.017601	1.926946
61	1	0	-11.693944	1.875223	4.201857
62	1	0	-7.846306	7.589572	2.452478
63	1	0	-8.422422	5.630036	5.033497
64	1	0	-9.921929	-1.861237	-4.134549
65	1	0	-7.759091	0.398695	-2.970571
66	1	0	-11.310791	0.877897	-7.44097
67	1	0	-8.186045	1.97372	-7.194637
68	1	0	-9.624433	5.856685	-5.490316
69	1	0	-12.708514	4.850925	-6.213711
70	1	0	-1.371039	0.888398	-7.502211
71	1	0	-9.311671	1.617144	7.062771
72	1	0	-8.415985	4.109287	10.690418
73	1	0	-4.991702	3.718088	10.267101
74	1	0	-5.024062	-3.305726	4.6125
75	1	0	-8.182093	-2.272112	4.740041
76	1	0	-6.531333	-2.987859	7.551729
77	1	0	-1.506195	-0.489331	5.990517
78	1	0	-2.063844	2.53721	7.243543

79	1	0	-2.880545	-0.187204	8.987739
80	1	0	5.573218	0.384844	8.394413
81	1	0	2.815807	-0.767495	6.945047
82	1	0	5.65237	-2.462021	6.671735
83	1	0	5.145416	4.540738	6.225541
84	1	0	2.263529	3.502768	4.974175
85	1	0	4.615324	4.761688	2.955974
86	1	0	12.058546	0.97239	4.94975
87	1	0	9.805636	-0.548679	7.111487
88	1	0	8.706703	2.40087	2.244519
89	1	0	-2.112803	-3.720153	-7.818854
90	1	0	14.903567	-9.313919	-0.365379
91	1	0	17.251202	-6.942982	-0.260941
92	1	0	17.81901	-8.544987	-4.536114
93	1	0	14.534853	-8.98337	-4.90494
94	1	0	15.079181	-4.921676	-6.956039
95	1	0	17.407456	-4.006214	-4.770628
96	1	0	7.683547	-4.292199	3.523363
97	1	0	6.704503	-6.310665	1.120776
98	1	0	7.434988	-3.135804	-3.207878
99	1	0	1.45498	2.203362	1.167283
100	1	0	4.252131	-6.616501	-2.970365
101	1	0	0.923729	-6.176731	-6.178429
6_tddft_		Standard Orientation (Ångstroms)			
Center number	Atomic number	Atomic Type	X	Y	Z
0	6	0	-3.248479	4.31597	-4.634769

1	6	0	-1.453865	2.37189	-4.5184
2	6	0	-1.24633	0.799473	-2.407669
3	6	0	-2.90962	1.308574	-0.416323
4	6	0	-4.786351	3.208947	-0.508104
5	6	0	-4.89848	4.739808	-2.659271
6	7	0	-3.09539	0.169373	1.888739
7	6	0	-5.041979	1.231475	3.285647
8	6	0	-6.161294	3.098049	1.832969
9	6	0	-5.42143	0.463681	6.023084
10	7	0	-11.321444	1.06861	1.571565
11	6	0	-10.896782	3.730988	1.093028
12	6	0	-8.442352	4.63408	2.4327
13	6	0	-10.824047	-0.818212	-0.037907
14	6	0	-10.790682	-0.165499	-2.792803
15	7	0	-10.856786	2.528698	-3.365228
16	6	0	-10.886849	4.405042	-1.683071
17	8	0	-10.634297	-3.052416	0.637985
18	8	0	-10.855655	6.669266	-2.304426
19	6	0	-8.56899	-1.0818	-4.371738
20	6	0	-8.928142	0.417687	-6.819412
21	6	0	-10.214957	2.904116	-6.035539
22	8	0	0.137451	2.115527	-6.550525
23	1	0	-12.453044	4.789973	1.926382
24	1	0	-12.516783	-1.017723	-3.552983
25	6	0	-8.066324	-0.529794	6.403486
26	6	0	-9.82976	0.377114	7.938762
27	6	0	-3.648384	-1.720201	6.746539

28	6	0	-4.799464	2.717231	7.727179
29	6	0	3.729795	1.625131	6.79122
30	6	0	6.196493	4.532374	3.991174
31	6	0	8.741834	0.184589	8.445486
32	6	0	8.395967	1.100983	6.139904
33	1	0	11.540918	0.459841	-5.301616
34	1	0	12.493508	-3.920722	1.390137
35	8	0	-1.253369	-3.702447	-5.527948
36	6	0	8.916028	-3.845837	-6.332618
37	6	0	7.366998	-1.607265	-7.35874
38	6	0	7.510339	0.457917	-5.332433
39	8	0	10.354027	-6.680722	-2.024277
40	8	0	10.593276	3.47201	-1.443174
41	6	0	10.40473	-4.335029	-1.949087
42	7	0	10.01535	-2.8923	-3.978115
43	6	0	10.011325	-0.139172	-4.043003
44	6	0	10.584656	1.134869	-1.579376
45	6	0	8.587833	-3.553643	2.396239
46	6	0	10.828492	-3.039569	0.562187
47	7	0	11.320295	-0.357523	0.324734
48	6	0	5.924307	1.77764	4.902272
49	6	0	6.218662	-2.18069	1.745823
50	6	0	5.295682	0.083177	2.677337
51	7	0	3.141051	0.703205	1.305701
52	6	0	4.355269	-5.100054	-1.78942
53	6	0	4.510488	-2.98276	-0.219556
54	6	0	2.626543	-1.10599	-0.452689

55	6	0	0.674264	-1.20526	-2.232601
56	6	0	0.614843	-3.356107	-3.763903
57	6	0	2.427374	-5.272408	-3.540357
58	1	0	-3.297657	5.497095	-6.300603
59	1	0	-6.250932	6.261915	-2.790602
60	1	0	-2.036194	-1.30209	2.448561
61	1	0	-11.310908	0.578637	3.421145
62	1	0	-8.171651	6.613122	1.931953
63	1	0	-8.839021	4.591608	4.444886
64	1	0	-8.599257	-3.112793	-4.656478
65	1	0	-6.813797	-0.566578	-3.432604
66	1	0	-10.140234	-0.62632	-8.11001
67	1	0	-7.146783	0.769106	-7.777789
68	1	0	-8.986864	4.537897	-6.195702
69	1	0	-11.911777	3.27726	-7.137275
70	1	0	1.411025	0.86401	-6.139357
71	1	0	-8.4805	-2.220236	5.315839
72	1	0	-11.643599	-0.55502	8.109026
73	1	0	-9.564298	2.053649	9.077891
74	1	0	-3.994194	-2.25919	8.699238
75	1	0	-1.669884	-1.171566	6.610414
76	1	0	-3.954121	-3.391397	5.584825
77	1	0	-4.945473	2.18911	9.710863
78	1	0	-2.872953	3.3474	7.379146
79	1	0	-6.0395	4.321184	7.401802
80	1	0	4.088772	2.834105	8.416042
81	1	0	1.979079	2.264609	5.927016

82	1	0	3.437724	-0.297164	7.460103
83	1	0	6.704104	5.74581	5.573438
84	1	0	4.440193	5.25652	3.200619
85	1	0	7.656576	4.698597	2.551832
86	1	0	10.631154	-0.134395	9.162389
87	1	0	7.204154	-0.304129	9.699737
88	1	0	10.059803	1.562526	5.038777
89	1	0	-2.50503	-2.378729	-5.327095
90	1	0	7.778123	-5.49713	-5.910557
91	1	0	10.40119	-4.413327	-7.638806
92	1	0	8.187578	-0.929537	-9.116331
93	1	0	5.430871	-2.167813	-7.75037
94	1	0	5.981144	0.283809	-3.969029
95	1	0	7.476469	2.353934	-6.114943
96	1	0	9.234041	-3.059557	4.277346
97	1	0	8.276672	-5.58866	2.400137
98	1	0	11.836758	0.545539	1.92422
99	1	0	2.11325	2.280927	1.54582
100	1	0	5.72805	-6.603622	-1.662639
101	1	0	2.273857	-6.905041	-4.758191

Table S3. The atom energies of the low-energy conformers of compound **1**.

Conformers	Gibbs free energies (ΔG) [a]	Final single point energy (a.u.)
1_tddft_	0.00000	-2406.554720381671
5_tddft_	0.00137	-2406.553355177897
6_tddft_	0.00134	-2406.553380015092

[a] CAM-B3LYP-D3/def2-SVP, in kcal/mol.

Table S4.Cartesian coordinates for the low-energy reoptimized random research conformers of compound **2** at PBE0-D3/def2-SVP level of theory in methanol.

8_tddft_		Standard Orientation (Ångstroms)			
Center number	Atomic number	Atomic Type	X	Y	Z
0	6	0	-1.599355	-0.505127	7.018406
1	6	0	-2.63135	0.646473	4.889392
2	6	0	-1.180445	0.932653	2.675219
3	6	0	1.234472	-0.085401	2.735331
4	6	0	2.275349	-1.307588	4.815193
5	6	0	0.84553	-1.468649	6.993646
6	7	0	3.040111	0.064928	0.829757
7	6	0	5.287053	-1.012793	1.504945
8	6	0	4.951584	-2.108866	4.193946
9	7	0	4.798878	3.117249	6.638121
10	6	0	7.207186	1.862678	6.220102
11	6	0	6.901851	-1.031155	6.057623
12	6	0	3.734016	4.929761	5.259182
13	6	0	5.380774	6.210503	3.349185
14	7	0	7.629952	4.775116	2.657199
15	6	0	8.677074	2.909429	3.986255
16	8	0	1.533403	5.666829	5.642211
17	8	0	10.756185	1.979273	3.448246
18	6	0	4.233773	6.872497	0.802466
19	6	0	6.586155	7.437518	-0.770531
20	6	0	8.599096	5.59673	0.191393
21	8	0	-5.038415	1.515613	5.07134

22	1	0	8.403638	2.198317	7.866755
23	1	0	5.976987	7.969986	4.265956
24	1	0	-10.252097	-3.596	0.517611
25	1	0	-9.89171	-1.417576	-6.37634
26	8	0	-2.48332	6.32815	2.333682
27	6	0	-6.854083	-7.38252	-1.297674
28	6	0	-6.627438	-7.054529	1.566878
29	6	0	-6.551091	-4.202077	2.001459
30	8	0	-7.565131	-5.861131	-6.345016
31	8	0	-8.198937	1.145254	1.005404
32	6	0	-7.795154	-4.283989	-4.631283
33	7	0	-7.701242	-4.897338	-2.190188
34	6	0	-8.296397	-3.242238	-0.066321
35	6	0	-8.220211	-0.483496	-0.693661
36	6	0	-6.061318	-0.593351	-7.133627
37	6	0	-8.130142	-1.514815	-5.307127
38	7	0	-8.35977	0.133979	-3.12352
39	6	0	-3.381182	-0.139607	-6.098961
40	6	0	-2.371555	-2.460605	-4.635666
41	7	0	-1.820244	-1.680865	-2.233624
42	6	0	-4.057716	4.390534	-4.075814
43	6	0	-3.300731	1.891655	-4.086191
44	6	0	-2.340915	0.874721	-1.861242
45	6	0	-2.091611	2.210127	0.383422
46	6	0	-2.752843	4.786468	0.2979
47	6	0	-3.759066	5.831817	-1.896507
48	8	0	7.154019	-1.065045	0.146691

49	6	0	5.118537	-5.121802	3.981094
50	6	0	4.817179	-6.348122	6.581527
51	6	0	7.640069	-6.004747	2.891719
52	6	0	2.911641	-5.987486	2.373445
53	6	0	2.929464	-6.457589	-0.090069
54	8	0	-2.047739	-4.61685	-5.397322
55	6	0	-1.419838	0.495892	-8.317738
56	6	0	-1.239536	-1.634533	-10.273699
57	6	0	1.197009	0.894323	-7.172755
58	6	0	-2.329454	2.800249	-9.716585
59	6	0	-1.247518	5.059445	-9.781138
60	1	0	-2.753332	-0.661352	8.695765
61	1	0	1.595163	-2.296382	8.702191
62	1	0	2.773467	0.852428	-0.881879
63	1	0	3.675887	2.348851	7.977685
64	1	0	8.766825	-1.778988	5.648127
65	1	0	6.404164	-1.684686	7.939866
66	1	0	2.934246	8.452982	0.921666
67	1	0	3.21196	5.257018	0.044408
68	1	0	7.187532	9.37616	-0.441954
69	1	0	6.255824	7.21323	-2.784117
70	1	0	8.80035	3.935916	-1.005493
71	1	0	10.449243	6.472906	0.382313
72	1	0	-5.913013	1.513169	3.428348
73	1	0	-1.149733	5.734963	3.481889
74	1	0	-5.057354	-7.814094	-2.202643
75	1	0	-8.194468	-8.850439	-1.822728

76	1	0	-8.274892	-7.854729	2.50094
77	1	0	-4.964563	-8.003653	2.308931
78	1	0	-4.653646	-3.465474	1.719459
79	1	0	-7.18364	-3.651833	3.871484
80	1	0	-6.730705	1.169283	-7.948122
81	1	0	-6.005045	-1.965146	-8.655357
82	1	0	-8.384875	2.008822	-3.485563
83	1	0	-0.9323	-2.812018	-0.982512
84	1	0	-4.868004	5.252691	-5.73725
85	1	0	-4.280696	7.806248	-1.886219
86	1	0	4.835853	-8.394339	6.357545
87	1	0	6.358417	-5.858088	7.849353
88	1	0	3.041294	-5.856201	7.483718
89	1	0	7.638839	-8.060337	2.793279
90	1	0	9.209792	-5.450694	4.096879
91	1	0	8.016776	-5.283606	1.013957
92	1	0	1.148779	-6.231144	3.392141
93	1	0	1.241634	-7.107444	-1.050047
94	1	0	4.59937	-6.258651	-1.25268
95	1	0	-3.021822	-1.981275	-11.235506
96	1	0	0.128716	-1.090919	-11.712391
97	1	0	-0.60952	-3.395737	-9.433623
98	1	0	2.54996	1.373985	-8.644449
99	1	0	1.882015	-0.820671	-6.265867
100	1	0	1.223056	2.41273	-5.788908
101	1	0	-4.019012	2.51321	-10.841475
102	1	0	-2.050519	6.566092	-10.909535

103	1	0	0.4413	5.526172	-8.728875
10_tddft_		Standard Orientation (Ångstroms)			
Center number	Atomic number	Atomic Type	X	Y	Z
0	6	0	-0.71577	-2.140656	6.991739
1	6	0	-2.129368	-0.72659	5.282592
2	6	0	-1.011255	0.19821	3.048322
3	6	0	1.480338	-0.499223	2.627312
4	6	0	2.90153	-1.975067	4.271432
5	6	0	1.792179	-2.753501	6.502835
6	7	0	2.993974	0.247879	0.609291
7	6	0	5.400318	-0.672894	0.779885
8	6	0	5.537513	-2.299183	3.20628
9	7	0	5.170181	2.267696	6.75495
10	6	0	7.604735	1.472036	5.758407
11	6	0	7.601181	-1.349212	5.019278
12	6	0	3.699901	4.141088	5.946143
13	6	0	4.868775	5.988957	4.151626
14	7	0	7.108102	5.033787	2.858312
15	6	0	8.570747	3.130391	3.622541
16	8	0	1.515872	4.460929	6.757788
17	8	0	10.631185	2.634774	2.630658
18	6	0	3.249614	6.978379	1.998917
19	6	0	5.231953	8.12654	0.243307
20	6	0	7.561271	6.433254	0.50841
21	8	0	-4.575307	-0.247008	5.891038
22	1	0	9.004971	1.642805	7.263354

23	1	0	5.426389	7.594389	5.336906
24	1	0	-9.613317	-5.036098	0.763776
25	1	0	-10.456331	-1.395165	-5.426076
26	8	0	-2.979886	5.277768	4.10368
27	6	0	-5.944386	-7.71924	-2.172589
28	6	0	-5.428588	-7.99222	0.659376
29	6	0	-5.712352	-5.33324	1.743953
30	8	0	-7.585349	-5.318905	-6.637243
31	8	0	-8.169542	-0.241417	2.158342
32	6	0	-7.785406	-4.19895	-4.592822
33	7	0	-7.251913	-5.283574	-2.382291
34	6	0	-7.810595	-4.250671	0.112122
35	6	0	-8.195686	-1.448033	0.138432
36	6	0	-6.889018	0.156614	-6.324723
37	6	0	-8.578029	-1.438575	-4.574958
38	7	0	-8.748449	-0.357306	-2.05624
39	6	0	-4.173314	0.762265	-5.486294
40	6	0	-2.687564	-1.639993	-4.726829
41	7	0	-1.921839	-1.330971	-2.276272
42	6	0	-5.170204	4.557506	-2.372984
43	6	0	-4.082161	2.285689	-3.064189
44	6	0	-2.711441	0.964473	-1.253273
45	6	0	-2.345673	1.793672	1.211016
46	6	0	-3.33708	4.191042	1.80567
47	6	0	-4.770771	5.515871	0.041635
48	8	0	7.058977	-0.243387	-0.76755
49	6	0	5.980156	-5.168347	2.356988

50	6	0	6.026325	-6.921614	4.656041
51	6	0	8.468074	-5.514641	0.940727
52	6	0	3.728405	-5.930341	0.762698
53	6	0	3.60239	-6.01133	-1.740361
54	8	0	-2.184219	-3.496486	-6.003506
55	6	0	-2.640747	2.140173	-7.70308
56	6	0	-2.591295	0.572696	-10.121113
57	6	0	0.090345	2.573486	-6.839226
58	6	0	-3.792689	4.707766	-8.15562
59	6	0	-5.278673	5.390733	-10.055373
60	1	0	-1.620729	-2.786784	8.704184
61	1	0	2.840488	-3.811121	7.898561
62	1	0	2.429658	1.335779	-0.845977
63	1	0	4.36333	1.096719	8.02986
64	1	0	9.459504	-1.738517	4.244865
65	1	0	7.451317	-2.416364	6.768258
66	1	0	1.854519	8.336725	2.638901
67	1	0	2.254182	5.424922	1.09023
68	1	0	5.6763	10.036239	0.863148
69	1	0	4.588791	8.232947	-1.703291
70	1	0	7.741461	5.074826	-1.025765
71	1	0	9.312125	7.505859	0.616287
72	1	0	-5.646667	-0.034368	4.385172
73	1	0	-1.445933	4.648197	4.939172
74	1	0	-4.221218	-7.625598	-3.294263
75	1	0	-7.100814	-9.246127	-2.919588
76	1	0	-6.823122	-9.238844	1.512792

77	1	0	-3.572688	-8.79319	1.020612
78	1	0	-3.98901	-4.238112	1.495138
79	1	0	-6.19504	-5.323326	3.735907
80	1	0	-7.84674	1.944914	-6.657287
81	1	0	-6.861733	-0.827724	-8.121505
82	1	0	-9.074911	1.522161	-1.969042
83	1	0	-0.76839	-2.581776	-1.418527
84	1	0	-6.336236	5.610707	-3.676237
85	1	0	-5.545605	7.328581	0.573895
86	1	0	4.257428	-6.890028	5.696116
87	1	0	6.319097	-8.85584	4.015567
88	1	0	7.558867	-6.463812	5.945518
89	1	0	8.612958	-7.466972	0.307186
90	1	0	10.076649	-5.15522	2.168541
91	1	0	8.647912	-4.29882	-0.6968
92	1	0	2.060509	-6.453778	1.836103
93	1	0	1.893123	-6.616644	-2.691385
94	1	0	5.173892	-5.523223	-2.953734
95	1	0	-1.71882	-1.255034	-9.808076
96	1	0	-4.453529	0.246698	-10.919262
97	1	0	-1.48619	1.569168	-11.542423
98	1	0	0.173003	3.673447	-5.102163
99	1	0	1.115236	3.627605	-8.277985
100	1	0	1.094846	0.79997	-6.553839
101	1	0	-3.277512	6.133984	-6.779223
102	1	0	-5.96484	7.313313	-10.199258
103	1	0	-5.878333	4.106535	-11.528809

Table S5. The atom energies of low-energy conformers of compound **2**.

Conformers	Gibbs free energies (ΔG) [a]	Final single point energy (a.u.)
8_tddft_	0.0000	-2556.903699068551
10_tddft_	0.0004	-2556.903301112253

[a] CAM-B3LYP-D3/def2-TZVP, in kcal/mol.

Table S6. Cartesian coordinates for the low-energy reoptimized random reseach conformers of comound **3** at PBE0-D3/def2-SVP level of theory in methanol.

2_tddft_		Standard Orientation (Ångstroms)			
Center number	Atomic number	Atomic Type	X	Y	Z
0	6	0	4.240471	1.518117	-2.434761
1	6	0	3.160243	2.276234	-0.146012
2	7	0	3.344619	4.62105	0.711118
3	6	0	4.64091	6.25574	-0.711634
4	6	0	5.761771	5.505642	-2.984991
5	7	0	5.539735	3.144969	-3.829769
6	7	0	4.850657	8.704158	0.1208
7	6	0	6.179905	10.500817	-1.22316
8	7	0	7.296647	9.738651	-3.484732
9	6	0	7.196697	7.322246	-4.499653
10	8	0	8.23369	6.781543	-6.50155
11	8	0	6.394838	12.688125	-0.494605
12	6	0	8.68087	11.705431	-4.845488
13	6	0	3.651349	9.425782	2.504491

14	6	0	4.071846	-1.136979	-3.400183
15	8	0	1.984554	-2.511978	-2.341082
16	6	0	-0.342208	-1.87591	-3.193103
17	6	0	-2.358634	-3.366249	-2.004518
18	8	0	-0.669646	-0.228223	-4.775731
19	6	0	-1.812033	-5.225101	-0.176289
20	6	0	-3.82319	-6.502501	0.820307
21	6	0	-6.293197	-6.021522	0.079817
22	6	0	-6.867938	-4.221278	-1.70528
23	6	0	-4.848434	-2.890474	-2.736696
24	8	0	-3.79682	-8.433734	2.549737
25	6	0	-6.376915	-8.919007	3.20363
26	8	0	-7.904182	-7.609072	1.314764
27	6	0	-6.910958	-11.692416	3.177134
28	6	0	-6.951641	-7.584419	5.738667
29	8	0	-9.229779	-8.211353	6.568091
30	6	0	-10.072061	-6.987594	8.869101
31	8	0	-5.516191	-6.113845	6.751713
32	1	0	2.121284	0.955986	1.017714
33	1	0	7.421679	13.25932	-5.299476
34	1	0	10.2275	12.401878	-3.691465
35	1	0	9.419867	10.893306	-6.567554
36	1	0	4.472204	8.361978	4.054897
37	1	0	3.964229	11.421006	2.801882
38	1	0	1.637799	9.049369	2.414501
39	1	0	3.969954	-1.134418	-5.450938
40	1	0	5.73115	-2.207021	-2.828821

41	1	0	0.096532	-5.630844	0.408947
42	1	0	-8.791828	-3.861491	-2.279856
43	1	0	-5.212689	-1.452492	-4.137263
44	1	0	-5.814725	-12.623923	4.645073
45	1	0	-8.902242	-12.03889	3.524503
46	1	0	-6.390298	-12.466331	1.347087
47	1	0	-8.812279	-7.461378	10.418445
48	1	0	-10.138043	-4.953795	8.600453
49	1	0	-11.947816	-7.726876	9.21897
3_tddft_		Standard Orientation (Ångstroms)			
Center number	Atomic number	Atomic Type	X	Y	Z
0	6	0	4.224626	1.545024	-2.334327
1	6	0	2.275535	3.327347	-2.234926
2	7	0	2.691632	5.742841	-1.730301
3	6	0	5.091066	6.425301	-1.333652
4	6	0	7.057991	4.664341	-1.467289
5	7	0	6.60312	2.236257	-1.953911
6	7	0	5.58037	8.915347	-0.79588
7	6	0	8.009353	9.783776	-0.409898
8	7	0	9.96108	8.019271	-0.548691
9	6	0	9.6751	5.462487	-1.058917
10	8	0	11.48353	4.014802	-1.156267
11	8	0	8.474776	12.006526	0.041342
12	6	0	12.499653	9.023096	-0.119474
13	6	0	3.477898	10.704747	-0.656646
14	6	0	3.745095	-1.175729	-2.946221

15	8	0	1.274003	-1.993846	-2.16146
16	6	0	0.922268	-2.27189	0.356068
17	6	0	-1.671156	-3.022822	0.997296
18	8	0	2.611443	-1.909675	1.887257
19	6	0	-3.539891	-3.29162	-0.881917
20	6	0	-5.899285	-3.97861	-0.086008
21	6	0	-6.456779	-4.39434	2.442968
22	6	0	-4.663589	-4.136051	4.307616
23	6	0	-2.248496	-3.439196	3.537944
24	8	0	-8.04604	-4.278543	-1.508229
25	6	0	-9.937072	-5.282881	0.147258
26	8	0	-8.950175	-4.986424	2.706747
27	6	0	-12.374118	-3.886817	-0.155621
28	6	0	-10.130753	-8.162477	-0.309034
29	8	0	-11.977847	-9.14364	1.068114
30	6	0	-12.312544	-11.857389	0.913108
31	8	0	-8.728202	-9.328993	-1.694767
32	1	0	0.334645	2.781771	-2.571204
33	1	0	12.610589	9.871355	1.744499
34	1	0	13.829875	7.479276	-0.2537
35	1	0	12.941921	10.439551	-1.535879
36	1	0	2.116917	10.071217	0.739543
37	1	0	4.221875	12.526588	-0.113462
38	1	0	2.556407	10.854525	-2.483514
39	1	0	3.750538	-1.461855	-4.981259
40	1	0	5.201681	-2.356297	-2.111317
41	1	0	-3.140062	-2.962784	-2.852466

42	1	0	-5.111291	-4.451459	6.272625
43	1	0	-0.78094	-3.217931	4.937489
44	1	0	-12.05275	-1.881665	0.152164
45	1	0	-13.104105	-4.173842	-2.05546
46	1	0	-13.761007	-4.571809	1.191223
47	1	0	-10.597082	-12.814926	1.506922
48	1	0	-13.852548	-12.285574	2.190648
49	1	0	-12.800854	-12.395279	-1.006552
4_tddft_		Standard Orientation (Ångstroms)			
Center number	Atomic number	Atomic Type	X	Y	Z
0	6	0	4.21574	1.438416	-2.700383
1	6	0	3.169277	2.2571	-0.417611
2	7	0	3.514955	4.577007	0.458999
3	6	0	4.942205	6.122464	-0.936781
4	6	0	6.027863	5.309076	-3.205614
5	7	0	5.644287	2.976965	-4.06959
6	7	0	5.322359	8.542781	-0.082307
7	6	0	6.787313	10.249215	-1.402299
8	7	0	7.879688	9.419574	-3.652111
9	6	0	7.606877	7.027091	-4.691823
10	8	0	8.617059	6.428541	-6.690911
11	8	0	7.141501	12.414448	-0.662053
12	6	0	9.422022	11.287592	-4.981026
13	6	0	4.152836	9.332121	2.294834
14	6	0	3.869871	-1.18541	-3.704567
15	8	0	1.806266	-2.502391	-2.536124

16	6	0	-0.546562	-1.777737	-3.233699
17	6	0	-2.533577	-3.214141	-1.934379
18	8	0	-0.915446	-0.09643	-4.770509
19	6	0	-1.93641	-5.167114	-0.223892
20	6	0	-3.922951	-6.379755	0.895379
21	6	0	-6.416869	-5.744254	0.388298
22	6	0	-7.041574	-3.850035	-1.279034
23	6	0	-5.046797	-2.586291	-2.435512
24	8	0	-3.855168	-8.376169	2.547863
25	6	0	-6.393124	-8.758349	3.403122
26	8	0	-8.001745	-7.290649	1.706462
27	6	0	-7.077843	-11.496653	3.313221
28	6	0	-6.67985	-7.506923	6.027506
29	8	0	-8.900723	-8.072445	7.036611
30	6	0	-9.480647	-6.903722	9.44465
31	8	0	-5.096505	-6.13729	6.958273
32	1	0	2.025955	1.009888	0.728421
33	1	0	8.274958	12.909014	-5.495165
34	1	0	10.965276	11.904636	-3.77897
35	1	0	10.171402	10.414956	-6.668682
36	1	0	2.113003	9.165514	2.161232
37	1	0	4.829997	8.15574	3.832501
38	1	0	4.660824	11.278133	2.644134
39	1	0	3.630316	-1.131455	-5.743586
40	1	0	5.521693	-2.329785	-3.272666
41	1	0	-0.010431	-5.690966	0.18318
42	1	0	-8.984535	-3.368486	-1.671211

43	1	0	-5.448735	-1.077615	-3.748722
44	1	0	-5.912847	-12.546684	4.641607
45	1	0	-9.047948	-11.756411	3.820829
46	1	0	-6.759203	-12.214306	1.415369
47	1	0	-8.111283	-7.487623	10.857486
48	1	0	-9.479434	-4.860206	9.252843
49	1	0	-11.349167	-7.577481	9.936423
5_tddft_		Standard Orientation (Ångstroms)			
Center number	Atomic number	Atomic Type	X	Y	Z
0	6	0	4.203413	0.701089	-4.204111
1	6	0	6.695306	0.052238	-3.62416
2	7	0	8.338864	1.701375	-2.704219
3	6	0	7.492071	4.049519	-2.321612
4	6	0	4.991508	4.718027	-2.877514
5	7	0	3.37516	3.040526	-3.823055
6	7	0	9.143076	5.803144	-1.363973
7	6	0	8.422556	8.273727	-0.92407
8	7	0	5.935325	8.928423	-1.49231
9	6	0	4.106954	7.302867	-2.436919
10	8	0	1.938658	8.001734	-2.864636
11	8	0	9.880945	9.855421	-0.070581
12	6	0	5.259437	11.563276	-1.004033
13	6	0	11.739814	5.04236	-0.795381
14	6	0	2.391585	-1.188557	-5.261225
15	8	0	0.261296	-1.600414	-3.595159
16	6	0	0.731417	-2.913332	-1.459904

17	6	0	-1.522629	-3.20031	0.138357
18	8	0	2.822802	-3.753407	-0.953451
19	6	0	-3.879299	-2.201977	-0.601251
20	6	0	-5.860397	-2.57842	1.013042
21	6	0	-5.592602	-3.866144	3.281666
22	6	0	-3.309737	-4.846186	4.044407
23	6	0	-1.26927	-4.4942	2.422692
24	8	0	-8.306754	-1.752187	0.761777
25	6	0	-9.708419	-2.870029	2.843511
26	8	0	-7.842382	-3.917636	4.533045
27	6	0	-11.262759	-0.922932	4.166819
28	6	0	-11.308682	-4.987689	1.633884
29	8	0	-10.083129	-7.168552	1.564396
30	6	0	-11.344866	-9.235209	0.285568
31	8	0	-13.391144	-4.594937	0.756168
32	1	0	7.36803	-1.85574	-3.915892
33	1	0	6.500472	12.807948	-2.059906
34	1	0	3.327222	11.848482	-1.600727
35	1	0	5.430361	11.974838	0.998844
36	1	0	12.728166	6.656378	-0.030907
37	1	0	11.735531	3.520007	0.578439
38	1	0	12.675163	4.409139	-2.507367
39	1	0	1.540075	-0.475681	-6.985867
40	1	0	3.324797	-2.973506	-5.652479
41	1	0	-4.115778	-1.1853	-2.350576
42	1	0	-3.111358	-5.841933	5.813567
43	1	0	0.55628	-5.243013	2.940332

44	1	0	-12.601943	-0.079895	2.861651
45	1	0	-12.305133	-1.795004	5.708235
46	1	0	-10.02753	0.53511	4.918798
47	1	0	-13.128697	-9.652667	1.210721
48	1	0	-11.659938	-8.753828	-1.68484
49	1	0	-10.067131	-10.825864	0.441498
6_tddft_		Standard Orientation (Ångstroms)			
Center number	Atomic number	Atomic Type	X	Y	Z
0	6	0	3.62957	0.842379	-5.075244
1	6	0	3.149029	2.732488	-6.853928
2	7	0	3.627001	5.150564	-6.413998
3	6	0	4.582461	5.721292	-4.147345
4	6	0	5.061776	3.843736	-2.343842
5	7	0	4.589089	1.422664	-2.828505
6	7	0	5.092528	8.208731	-3.626951
7	6	0	6.083152	8.957966	-1.330921
8	7	0	6.560359	7.079275	0.452578
9	6	0	6.112116	4.516052	0.126514
10	8	0	6.568371	2.963779	1.786541
11	8	0	6.540926	11.17373	-0.843191
12	6	0	7.607579	7.960614	2.853419
13	6	0	4.572151	10.12399	-5.552036
14	6	0	3.079233	-1.873292	-5.613874
15	8	0	1.166591	-2.87013	-3.929166
16	6	0	-1.22188	-2.07493	-4.334264
17	6	0	-3.026548	-3.127744	-2.503314

18	8	0	-1.777569	-0.636493	-6.054608
19	6	0	-2.224851	-4.743128	-0.542083
20	6	0	-4.048092	-5.612247	1.067677
21	6	0	-6.573497	-4.955432	0.807945
22	6	0	-7.398076	-3.391163	-1.095991
23	6	0	-5.570899	-2.479899	-2.754192
24	8	0	-3.771582	-7.239246	3.07065
25	6	0	-6.213134	-7.325807	4.326168
26	8	0	-7.973559	-6.109864	2.635419
27	6	0	-6.995516	-9.989928	4.822526
28	6	0	-5.874092	-5.753362	6.760604
29	8	0	-6.266347	-3.314835	6.3578
30	6	0	-5.792362	-1.63678	8.469153
31	8	0	-5.198909	-6.690177	8.742343
32	1	0	2.355933	2.266962	-8.67912
33	1	0	9.392071	8.917831	2.528937
34	1	0	6.298078	9.256496	3.755957
35	1	0	7.905559	6.33634	4.054741
36	1	0	5.669647	9.734405	-7.240028
37	1	0	2.576401	10.118112	-6.023551
38	1	0	5.094568	11.948148	-4.799098
39	1	0	2.499399	-2.143637	-7.563333
40	1	0	4.728942	-3.029921	-5.229692
41	1	0	-0.270698	-5.272614	-0.315871
42	1	0	-9.365754	-2.89007	-1.291268
43	1	0	-6.132296	-1.235161	-4.269718
44	1	0	-5.613732	-10.922275	6.018129

45	1	0	-8.814754	-10.015699	5.777394
46	1	0	-7.147549	-10.99588	3.038906
47	1	0	-7.047416	-2.095219	10.027053
48	1	0	-3.833663	-1.789131	9.063373
49	1	0	-6.185527	0.242758	7.761521
7_tddft_		Standard Orientation (Ångstroms)			
Center number	Atomic number	Atomic Type	X	Y	Z
0	6	0	4.555816	-0.068539	-3.916376
1	6	0	7.110096	-0.443522	-3.364997
2	7	0	8.635837	1.42968	-2.710288
3	6	0	7.60762	3.73215	-2.572783
4	6	0	5.047531	4.129039	-3.110093
5	7	0	3.548506	2.226124	-3.786003
6	7	0	9.132767	5.711704	-1.881386
7	6	0	8.22695	8.154111	-1.721006
8	7	0	5.681019	8.538934	-2.283947
9	6	0	3.96448	6.670958	-2.947261
10	8	0	1.732678	7.138465	-3.365168
11	8	0	9.571233	9.937878	-1.114456
12	6	0	4.805105	11.153419	-2.094869
13	6	0	11.796452	5.228059	-1.314898
14	6	0	2.881825	-2.214359	-4.671953
15	8	0	0.693861	-2.425835	-3.048732
16	6	0	1.133075	-3.222748	-0.665955
17	6	0	-1.184149	-3.362753	0.861162
18	8	0	3.246986	-3.765307	0.091395

19	6	0	-3.554695	-2.699117	-0.157319
20	6	0	-5.595721	-2.909796	1.410764
21	6	0	-5.375256	-3.722745	3.893793
22	6	0	-3.080157	-4.368889	4.928278
23	6	0	-0.97682	-4.178618	3.360354
24	8	0	-8.072641	-2.346153	0.893893
25	6	0	-9.520203	-3.090635	3.069072
26	8	0	-7.684223	-3.703852	5.035972
27	6	0	-11.209066	-0.995069	3.911813
28	6	0	-10.946933	-5.582452	2.540522
29	8	0	-9.591481	-7.126509	1.11166
30	6	0	-10.661449	-9.600324	0.626413
31	8	0	-13.004794	-6.050157	3.437766
32	1	0	7.931954	-2.313172	-3.455434
33	1	0	2.855185	11.221762	-2.69826
34	1	0	4.947025	11.808626	-0.15523
35	1	0	5.944954	12.35762	-3.300413
36	1	0	12.753847	4.524491	-2.987051
37	1	0	12.656031	6.981166	-0.719756
38	1	0	11.948042	3.8415	0.187677
39	1	0	2.089239	-1.88867	-6.53619
40	1	0	3.914661	-3.987373	-4.679613
41	1	0	-3.759595	-2.05498	-2.078973
42	1	0	-2.918472	-4.993937	6.863417
43	1	0	0.860867	-4.676181	4.093472
44	1	0	-12.21507	-1.534014	5.616645
45	1	0	-10.075311	0.676026	4.284282

46	1	0	-12.583449	-0.570654	2.444933
47	1	0	-10.940795	-10.604256	2.394781
48	1	0	-12.443071	-9.410971	-0.374409
49	1	0	-9.282746	-10.565259	-0.537802
8_tddft_		Standard Orientation (Ångstroms)			
Center number	Atomic number	Atomic Type	X	Y	Z
0	6	0	4.923385	-0.389809	-3.36952
1	6	0	7.287579	-0.746442	-2.251815
2	7	0	8.706192	1.159017	-1.461064
3	6	0	7.75533	3.478677	-1.76232
4	6	0	5.386051	3.860636	-2.880597
5	7	0	3.997069	1.92377	-3.679753
6	7	0	9.166528	5.493139	-0.945069
7	6	0	8.331096	7.953216	-1.204178
8	7	0	5.97859	8.320058	-2.330103
9	6	0	4.392058	6.423328	-3.205572
10	8	0	2.335532	6.884155	-4.169537
11	8	0	9.578571	9.766391	-0.488332
12	6	0	5.175266	10.953269	-2.569187
13	6	0	11.624796	5.029115	0.231737
14	6	0	3.350043	-2.568569	-4.233757
15	8	0	1.012136	-2.743922	-2.8209
16	6	0	1.204321	-3.571124	-0.415934
17	6	0	-1.241671	-3.618557	0.900294
18	8	0	3.217797	-4.203197	0.5236
19	6	0	-3.478891	-2.816695	-0.303228

20	6	0	-5.65441	-2.939072	1.084229
21	6	0	-5.689182	-3.798237	3.561533
22	6	0	-3.528308	-4.580658	4.774547
23	6	0	-1.292725	-4.479583	3.392947
24	8	0	-8.044151	-2.218174	0.376535
25	6	0	-9.709072	-2.968747	2.42859
26	8	0	-8.083204	-3.673594	4.501709
27	6	0	-11.401982	-0.838985	3.173861
28	6	0	-11.159476	-5.295018	1.430089
29	8	0	-9.882589	-7.416541	1.797949
30	6	0	-10.980177	-9.697713	0.755772
31	8	0	-13.165383	-5.114529	0.333475
32	1	0	8.043853	-2.627588	-1.994583
33	1	0	5.029607	11.814355	-0.713006
34	1	0	6.53342	11.99925	-3.695444
35	1	0	3.351132	10.983877	-3.486684
36	1	0	11.393945	3.816216	1.868775
37	1	0	12.88668	4.125227	-1.109345
38	1	0	12.408221	6.823068	0.80993
39	1	0	2.73983	-2.29165	-6.17201
40	1	0	4.383047	-4.335412	-4.089676
41	1	0	-3.483317	-2.130567	-2.221418
42	1	0	-3.564892	-5.242622	6.703663
43	1	0	0.447862	-5.086053	4.267143
44	1	0	-12.538857	-0.247823	1.571982
45	1	0	-12.65742	-1.440184	4.685386
46	1	0	-10.260184	0.739666	3.823229

47	1	0	-12.812703	-10.051467	1.609959
48	1	0	-11.166765	-9.521501	-1.280834
49	1	0	-9.666799	-11.193372	1.230578
9_tddft_		Standard Orientation (Ångstroms)			
Center number	Atomic number	Atomic Type	X	Y	Z
0	6	0	2.361419	2.234714	-5.177862
1	6	0	4.748204	1.637326	-6.132855
2	7	0	6.823131	2.851441	-5.432985
3	6	0	6.53707	4.688263	-3.72412
4	6	0	4.155544	5.290596	-2.737082
5	7	0	2.090431	4.06638	-3.481982
6	7	0	8.648181	5.97821	-2.951571
7	6	0	8.517929	7.895355	-1.184378
8	7	0	6.141578	8.491299	-0.217573
9	6	0	3.891117	7.307203	-0.860169
10	8	0	1.863308	7.919578	0.08099
11	8	0	10.392096	9.051683	-0.471875
12	6	0	6.095971	10.534281	1.642921
13	6	0	11.107853	5.301326	-4.017677
14	6	0	0.04635	0.848491	-6.009113
15	8	0	-1.162819	-0.442084	-3.923038
16	6	0	0.025709	-2.515338	-3.033274
17	6	0	-1.277591	-3.64446	-0.854662
18	8	0	1.975105	-3.327667	-3.969376
19	6	0	-3.451587	-2.516936	0.192316
20	6	0	-4.52146	-3.711335	2.217624

21	6	0	-3.530201	-5.921775	3.222771
22	6	0	-1.406296	-7.049326	2.234734
23	6	0	-0.292028	-5.867979	0.166335
24	8	0	-6.574754	-2.966817	3.614498
25	6	0	-7.111401	-4.983855	5.336987
26	8	0	-4.917	-6.655746	5.267345
27	6	0	-7.528911	-3.984125	7.945341
28	6	0	-9.328346	-6.551697	4.257894
29	8	0	-10.086483	-8.264919	5.919645
30	6	0	-12.082542	-9.955343	5.108821
31	8	0	-10.174611	-6.268103	2.147724
32	1	0	4.97575	0.13402	-7.499039
33	1	0	4.157619	10.842518	2.206963
34	1	0	7.217328	10.017255	3.281207
35	1	0	6.857208	12.249779	0.815908
36	1	0	12.519154	6.533705	-3.207477
37	1	0	11.553998	3.350591	-3.56692
38	1	0	11.074387	5.536377	-6.054341
39	1	0	-1.379927	2.165407	-6.671545
40	1	0	0.481093	-0.489853	-7.502647
41	1	0	-4.232944	-0.789728	-0.553293
42	1	0	-0.639474	-8.765063	3.028088
43	1	0	1.372852	-6.693763	-0.675198
44	1	0	-7.792861	-5.528224	9.26932
45	1	0	-5.901432	-2.866101	8.51162
46	1	0	-9.209306	-2.800871	7.972896
47	1	0	-11.469879	-11.038715	3.476432

48	1	0	-12.423588	-11.192067	6.702948
49	1	0	-13.772695	-8.881999	4.657523
10_tddft_		Standard Orientation (Ångstroms)			
Center number	Atomic number	Atomic Type	X	Y	Z
0	6	0	4.706044	-0.145528	-3.724174
1	6	0	7.215532	-0.578372	-3.030518
2	7	0	8.744335	1.259913	-2.287997
3	6	0	7.760482	3.583966	-2.199467
4	6	0	5.241335	4.037533	-2.872498
5	7	0	3.742499	2.170516	-3.64089
6	7	0	9.289552	5.529933	-1.427016
7	6	0	8.42105	7.987027	-1.284614
8	7	0	5.910604	8.423916	-1.95246
9	6	0	4.20196	6.599077	-2.744977
10	8	0	2.008937	7.117488	-3.289057
11	8	0	9.769943	9.740595	-0.604109
12	6	0	5.073242	11.050784	-1.767116
13	6	0	11.913226	4.99251	-0.739707
14	6	0	3.032554	-2.250789	-4.585435
15	8	0	0.755126	-2.435897	-3.087885
16	6	0	1.046511	-3.248249	-0.687853
17	6	0	-1.354434	-3.362067	0.704303
18	8	0	3.106952	-3.819616	0.186617
19	6	0	-3.660757	-2.709681	-0.458763
20	6	0	-5.789805	-2.8979	0.991542
21	6	0	-5.7146	-3.678215	3.493894

22	6	0	-3.484905	-4.310758	4.669369
23	6	0	-1.294748	-4.144273	3.222664
24	8	0	-8.231762	-2.327139	0.327558
25	6	0	-9.801437	-3.097724	2.447131
26	8	0	-8.086746	-3.649545	4.495025
27	6	0	-11.586147	-1.037596	3.173397
28	6	0	-11.149154	-5.528063	1.559845
29	8	0	-9.754738	-7.567337	1.96185
30	6	0	-10.759272	-9.936483	1.026646
31	8	0	-13.189062	-5.487241	0.512529
32	1	0	7.998815	-2.466002	-3.076778
33	1	0	6.222944	12.238542	-2.981139
34	1	0	3.121554	11.144262	-2.361282
35	1	0	5.235967	11.704353	0.170626
36	1	0	12.923354	4.241181	-2.359105
37	1	0	12.788054	6.734405	-0.133861
38	1	0	11.967625	3.625685	0.787922
39	1	0	2.347484	-1.8898	-6.48588
40	1	0	4.02953	-4.044228	-4.559321
41	1	0	-3.750679	-2.089696	-2.397176
42	1	0	-3.437102	-4.910646	6.618473
43	1	0	0.496544	-4.635788	4.066277
44	1	0	-12.79698	-0.560866	1.587345
45	1	0	-12.765615	-1.65516	4.738741
46	1	0	-10.515265	0.620434	3.739959
47	1	0	-9.357808	-11.345881	1.513276
48	1	0	-12.547758	-10.353637	1.943185

49	1	0	-11.008094	-9.839157	-1.00852

Table S7. The atom energies of low-energy conformers of comound **3**.

Conformers	Gibbs free energies (ΔG) [a]	Final single point energy (a.u.)
2_tddft_	0.00028	-1593.792780676491
3_tddft_	0.00007	-1593.792987083299
4_tddft_	0.00032	-1593.792735343297
5_tddft_	0.00016	-1593.792902050109
6_tddft_	0.0002	-1593.792862655066
7_tddft_	0.00028	-1593.792774643712
8_tddft_	0.00012	-1593.792934170733
9_tddft_	0.00000	-1593.793058492482
10_tddft_	0.00015	-1593.792905237641

[a] CAM-B3LYP-D3/def2-TZVP, in kcal/mol.
